# Sall2 is required for proapoptotic Noxa expression and genotoxic stress-induced apoptosis by doxorubicin

**DOI:** 10.1038/cddis.2015.165

**Published:** 2015-07-16

**Authors:** D Escobar, M I Hepp, C Farkas, T Campos, N M Sodir, M Morales, C I Álvarez, L Swigart, G I Evan, J L Gutiérrez, R Nishinakamura, A F Castro, R Pincheira

**Affiliations:** 1 Departamento de Bioquímica y Biología Molecular, Facultad de Ciencias Biológicas, Universidad de Concepción, Concepción, Chile; 2 Department of Biochemistry, University of Cambridge, Cambridge, UK; 3 Helen Diller Family Comprehensive Cancer Center, University of California San Francisco, San Francisco, CA 94115, USA; 4 Department of Kidney Development, Institute of Molecular Embryology and Genetics, Kumamoto University, Kumamoto, Japan

## Abstract

The Sall2 transcription factor is deregulated in several cancers; however, little is known about its cellular functions, including its target genes. Recently, we demonstrated that p53 directly regulates Sall2 expression under genotoxic stress. Here, we investigated the role of Sall2 in the context of cellular response to genotoxic stress. In addition, we further examined the Sall2-p53 relationship during genotoxic stress in primary mouse embryo fibroblasts (MEFs), which are derived from *Sall2* knockout mice separately, or in combination with the *p53ERTAM* knock-in mice. We found that the levels of Sall2 mRNA and protein are dynamically modulated in response to doxorubicin. At early times of stress, Sall2 is downregulated, but increases under extension of the stress in a p53-independent manner. Based on caspase-3/7 activities, expression of cleaved poly (ADP-ribose) polymerase, expression of cleaved caspase-3 and induction of proapoptotic proteins, Sall2 expression was correlated with cellular apoptosis. Consequently, *Sall2*^*−/−*^ MEFs have decreased apoptosis, which relates with increased cell viability in response to doxorubicin. Importantly, Sall2 was required for apoptosis even in the presence of fully activated p53. Searching for putative Sall2 targets that could mediate its role in apoptosis, we identified proapoptotic *NOXA/PMAIP1* (phorbol-12-myristate-13-acetate-induced protein 1). We demonstrated that Sall2 positively regulates *Noxa* promoter activity. Conserved putative Sall2-binding sites at the *NOXA* promoter were validated *in vitro* by electrophoretic mobility shift assay and *in vivo* by ChIP experiments, identifying *NOXA* as a novel Sall2 target. In agreement, induction of Noxa protein and mRNA in response to doxorubicin was significantly decreased in *Sall2*^*−/−*^ MEFs. In addition, studies in leukemia Jurkat T cells support the existence of the Sall2/Noxa axis, and the significance of this axis on the apoptotic response to doxorubicin in cancer cells. Our study highlights the relevance of Sall2 in the apoptotic response to extended genotoxic stress, which is important for understanding its role in normal physiology and disease.

Deregulation of the Sall2 transcription factor is associated with the development of human diseases, including cancer, ocular coloboma and Alport syndrome.^1, 2, 3, 4^ However, Sall2 normal function, regulation and immediate target genes are not well known, making it difficult to understand its role in various diseases.

*SALL2* is a member of the Spalt/Sal family of transcription factors characterized by their role in organ development and conserved from *Caenorhabditis elegans* to humans.^5, 6, 7^ *Sall2*-deficient mice were previously reported to have no apparent abnormal phenotype when bred on a C57BL/6 genetic background; however, a strain-specific incidence of neural tube defects and perinatal lethality were reported when bred on mixed genetic backgrounds.^8, 9^ Recently, it was demonstrated that Sall2 has a role in eye morphogenesis and a *SALL2* gene mutation was associated with coloboma, a congenital eye defect.^2, 10^ A deleterious *SALL2* mutation was also associated with Alport syndrome, a renal disease,^1^ suggesting that Sall2 could have a role in kidney development.

Evidences for Sall2 association with cancer are increasing, but are still controversial. Several studies suggest a tumor suppressor role for Sall2 in ovarian cancer^3, 11, 12^ and in primary acute myeloid leukemia.^13^ However, Sall2 is found upregulated in Wilm's tumor,^14^ synovial sarcoma,^15, 16^ oral cancer^17, 18^ and testicular cancer,^19^ and is one of the four neurodevelopmental transcription factors essential for glioblastoma propagation.^4^ The molecular mechanisms underlying the role of Sall2 as a tumor suppressor in certain types of cancer and its deregulation in others are still unknown.

To understand the role of Sall2 in normal and disease states, it is essential to define Sall2 targets under different cell contexts. Sall2 targets identified to date include the cell cycle regulatory gene *p21*^*WAF*^ (protein 21 wild-type p53 activation factor), the proapoptotic gene *BAX* (B-cell lymphoma 2 (BCL2)-associated X protein) and the proto-oncogene *c-Myc*. Although Sall2 upregulates p21^WAF^ and BAX, it represses c-Myc.^12, 20, 21^ Sall2 targets were identified in a p53-independent context, with Sall2 and p53 having common targets. The *p53* gene is mutated in over 50% of human cancers (http://www-p53.iarc.fr/). In response to various types of stress, signaling pathways converge to induce transcriptional regulation by p53 of target genes involved in cellular responses including cell cycle arrest, apoptosis, senescence, autophagy, DNA repair and central metabolism.^22, 23^ p53 prevents tumor formation through transcriptional-dependent and -independent mechanisms.^24, 25, 26^ We recently demonstrated that *SALL2* is also a p53 target gene. At early times of genotoxic stress, p53 downregulates Sall2 by directly binding to the *SALL2* promoter.^27^ Here, we investigated the role of Sall2 in the cellular response to doxorubicin and further examined the Sall2-p53 relationship during genotoxic stress in mouse embryonic fibroblasts (MEFs) derived from *Sall2* knockout mice separately or in combination with the *p53ERTAM* knock-in mice. We show that Sall2 is required for full apoptotic response to doxorubicin, and demonstrate that apoptosis is significantly decreased in Sall2-deficient cells even in the presence of activated p53. More importantly, we identified Noxa, a member of the Bcl-2 homology domain 3 (BH3)-only proteins,^28^ as a novel Sall2 target. We demonstrate that Sall2 binds to and transactivates the *NOXA* promoter under genotoxic stress *in vitro* and *in vivo*. Finally, we show that the Sall2/Noxa axis is also important for the cell death response to doxorubicin in Jurkat T cells, supporting the significance of this axis in a cancer cell context. This new data supports the tumor suppressor function of Sall2, and enhances the understanding of Sall2 role in the context of genotoxic stress.

## Results

### Sall2 expression is dynamically regulated during doxorubicin treatment

We recently demonstrated that Sall2 is downregulated by p53 during genotoxic stress.^27^ To understand the role of Sall2 during this stress, we used primary Sall2 wild-type (*Sall2*^*+/+*^) and Sall2 knockout^9^ (*Sall2*^*−/−*^) MEFs, and primary MEFs derived from *Sall2*^*+/*^^*−*^ model crossed with a conditional *p53ERTAM* knock-in (*p53*^*ER/ER*^) model described previously.^29^ *p53*^*ER/ER*^ mice and cells derived from it can be reversibly and rapidly switched between p53 wild-type and knockout states by, respectively, administration or withdrawal of 4-hydroxytamoxifen (4-OHT).^29, 30, 31, 32^ We used these cells to investigate p53-dependent and -independent Sall2 responses.

**Figure 1:**
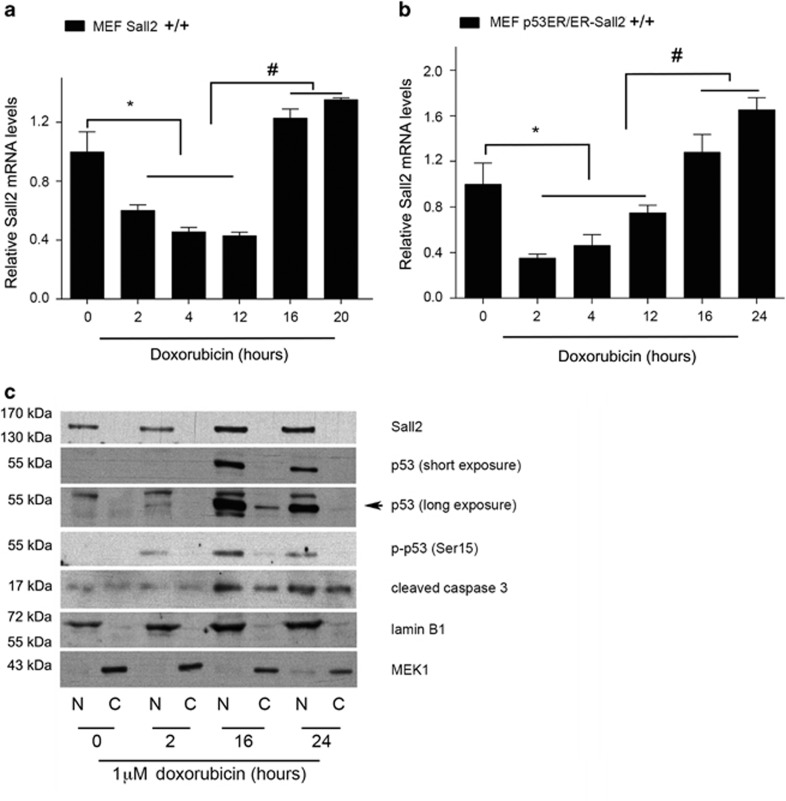
Dynamic regulation of Sall2 under genotoxic stress induced by doxorubicin. Wild-type *Sall2*^+/+^ (**a**) or *p53*^*ER/ER*^*; Sall2*^+/+^ (**b**) MEFs were exposed to 1 *μ*M doxorubicin for various times. In (**b**), before doxorubicin treatment, *p53*^*ER/ER*^*; Sall2* ^+/+^ MEFs were treated for 4 h with 4-OHT 100 nM to allow p53 functionality. Sall2 mRNA levels were measured by quantitative real-time PCR (qPCR) relative to *cyclophilin A*. Values from triplicate samples are representative of three independent experiments. Expression at time 0 was defined as 1. Each bar represents the mean±S.D. *P<0.05 for decrease of Sall2 relative to *t*=0 h; ^#^*P*<0.05 for increase of Sall2 relative to *t*=12 h. (**c**) *Sall2*^*+/+*^ MEFs were exposed to 1 *μ*M doxorubicin for the indicated times. Lysates were collected and subcellular fractionation was performed to obtain nuclear (N) and cytoplasmic (C) fractions. Levels of Sall2, total and activated (Ser18) p53 and cleaved caspase-3 were determined by western blot using specific antibodies. Lamin B1 and MEK1 are nuclear and cytoplasmic markers, respectively. The figure is representative of two independent experiments

We treated *Sall2*^*+/+*^ MEFs and *p53*^*ER/ER*^; *Sall2*^*+/+*^ MEFs (in the presence of 4-OHT for p53 wild-type state) with 1 *μ*M doxorubicin and isolated RNA after different times of treatment. Consistent with our previous report, we observed a dynamic regulation of Sall2 expression over the treatment period. Sall2 mRNA levels significantly decreased after 2 h, but they recovered and increased after 12 h in both cell models (Figures 1a and b). The changes on Sall2 expression were also observed using a lower dose of doxorubicin (0.5 *μ*M) (Supplementary Figure 1).

To determine if the increase of Sall2 mRNA levels correlates with the expression of potentially functional Sall2, we performed subcellular fractionation of MEFs exposed to 1 *μ*M doxorubicin and evaluated Sall2 protein expression and location. According to the mRNA results, Sall2 protein increased at 16 h and localized exclusively at the cell nucleus (Figure 1c). As expected, doxorubicin induced p53 activation,^33, 34, 35^ evidenced by an increase on p53 levels and its phosphorylation at Ser18 (p-p53) in the nucleus. Doxorubicin mainly induces cell cycle arrest in MEFs;^33, 34, 36, 37^ still, several reports indicate that extended doxorubicin treatment (using 1 *μ*M or higher concentration) could result in apoptosis.^38, 39, 40^ We detected nuclear cleaved caspase-3 between 16 and 24 h of doxorubicin treatment. As caspase-3 is cleaved during apoptosis and is translocated into the nucleus to cleave its substrates,^41, 42^ our results suggest that the Sall2 upregulation correlates with apoptosis.

### Sall2 expression is required for doxorubicin-induced apoptosis of MEFs

**Figure 2:**
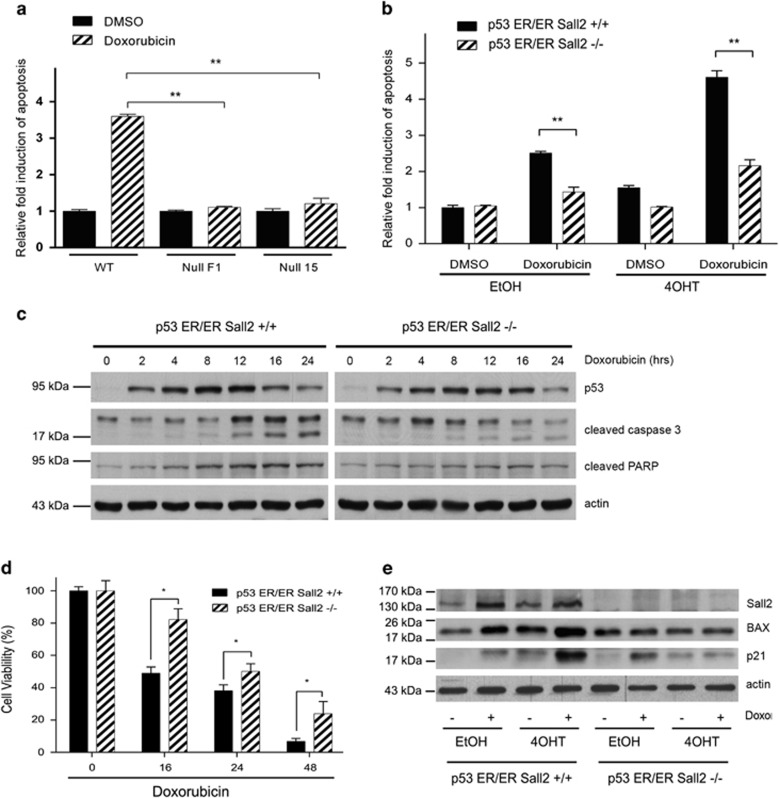
Sall2 expression is required for doxorubicin-induced apoptosis of primary MEFs. (**a**) MEFs *Sall2* ^*+/+*^ (wild-type (WT)) and *Sall2*^*−/−*^ (null F1 and 15) were exposed to 1 *μ*M Doxorubicin or vehicle (DMSO) for 16 h. Cellular apoptosis was measured by using a caspase-3/7 glow assay. Values are plotted as the mean±S.D. of quintuplicate exposures to drug or vehicle from three independent experiments. Relative fold induction of apoptosis was normalized to caspase-3/7 activity of *Sall2*^*+/+*^ MEFs exposed to DMSO (**P<0.001). (**b**) *p53*^*ER/ER*^*; Sall2*^*+/+*^ and *p53*^*ER/ER*^*;* *Sall2*^*−/−*^ MEFs treated with 4-OHT 100 nM for 4 h (for functional p53) and then exposed to 1 *μ*M doxorubicin or vehicle (DMSO) for 16 h. Apoptosis was measured as in (**a**). A significant difference in doxorubicin-dependent apoptosis between *Sall2*^*+/+*^ and *Sall2*^*−/−*^ MEFs was noted in the absence (EtOH) and presence (4-OHT) of functional p53 (**P<0.001). (**c**) *p53*^*ER/ER*^; *Sall2*^*+/+*^ and *Sall2*^*−/−*^ MEFs were treated for 4 h with 4-OHT and then exposed to 1 *μ*M doxorubicin. Cell lysates were collected at various times and levels of total p53 (detected as p53ER fusion protein), cleaved caspase-3 and cleaved PARP were analyzed by western blot. Actin shows equal loading. (**d**) *p53*^*ER/ER*^*; Sall2*^*+/+*^ and *p53*^*ER/ER*^*;* *Sall2*^*−/−*^ MEFs treated for 4 h with 4-OHT 100 nM and then exposed to 1 *μ*M doxorubicin or vehicle (DMSO) for 16, 24 and 48 h. Cellular viability was measured using a CytoTox-Glo kit assay. Cell viability (%) is plotted as the mean±S.D. of two independent experiments performed in triplicate. Results are expressed as the percentage of survival relative to control (0- h time point). A significant difference in doxorubicin-dependent cellular viability between *Sall2*^*+/+*^ and *Sall2*^*−/−*^ MEFs was noted at each time evaluated (16, 24 and 48 h, *P<0.05). (**e**) *p53*^*ER/ER*^*; Sall2*^*+/+*^ and *p53*^*ER/ER*^*; Sall2*^*−/−*^ MEFs were treated for 4 h with 4-OHT 100 nM or EtOH and then exposed to 1 *μ*M doxorubicin for 16 h. Western blot evaluated levels of Sall2, BAX and p21. Actin shows equal loading. Figures are representative of three independent experiments

To determine whether Sall2 contributes to apoptosis under genotoxic stress, we exposed *Sall2*^*+/+*^ and *Sall2*^*−/−*^ MEFs to doxorubicin (1 *μ*M) for 16 h, and then examined apoptosis through caspase-3/7 activity. Figure 2a shows that *Sall2*^*−/−*^ cells are more resistant to apoptosis compared with *Sall2*^+/+^ MEFs. *Sall2*^*+/+*^ MEFs showed a statistically significant increase in caspase-3/7 activity (>3-fold) compared with untreated control cells, whereas two independent *Sall2*^*−/−*^ MEFs had a slight increase in apoptosis. This indicates that Sall2-competent cells are more prone to apoptosis induced by genotoxic stress compared with Sall2-deficient cells.

Because of the important role of p53 in genotoxic stress-induced apoptosis, we analyzed whether the role of Sall2 is dependent on p53 using *Sall2*^*+/+*^ or *Sall2*^*−/−*^ MEFs in combination with *p53*^*ER/ER*^ MEFs. In the cell model, p53 is expressed in the presence of ethanol (EtOH) but remains inactive even when an activating signal such as doxorubicin is added; in the presence of 4-OHT, p53 is expressed and has the capacity to be activated by doxorubicin but not by the addition of dimethyl sulfoxide (DMSO).^29^ We exposed conditional *p53*^*ER/ER*^; *Sall2*^*+/+*^ and *p53*^*ER/ER*^; *Sall2*^*−/−*^ MEFs to doxorubicin, and measured apoptosis as above. The *p53*^*ER/ER*^; *Sall2*^*−/−*^ MEFs also showed significantly lower levels of apoptosis compared with the *p53*^*ER/ER*^; *Sall2*^*+/+*^ MEFs either in the absence (EtOH) or presence (4-OHT) of fully active p53 (Figure 2b, compare bars 3–4 and 7–8). The residual apoptotic activity in the absence of 4-OHT may indicate that Sall2 affects apoptosis independently of p53. However, the apoptotic response is significantly increased when p53 and Sall2 are present (Figure 2b; >4.5-fold, bar 7). Additionally, we analyzed the expression of apoptotic markers over the time of treatment with doxorubicin in *p53*^*ER/ER*^; *Sall2*^*+/+*^ and *p53*^*ER/ER*^; *Sall2*^*−/−*^ MEFs treated with 4-OHT. The results shown in Figure 2c indicate that in response to doxorubicin, p53 increases in both *Sall2*^*+/+*^ and *Sall2*^*−/−*^ MEFs. However, apoptosis is decreased in the *Sall2*^*−/−*^ MEFs, evidenced by the low levels of cleaved caspase-3 and poly (ADP-ribose) polymerase (PARP), especially after 12 h of treatment. Consistent with a role for Sall2 in cell death response, cell viability was increased in *Sall2*^*−/−*^ MEFs between 16-48 h of doxorubicin treatment (Figure 2d), which correlated with the decreased apoptotic response observed in Figures 2a–c. Taken together, our data indicate that Sall2 is required for the doxorubicin-induced p53-dependent apoptosis of MEFs, but Sall2 may induce apoptosis independently of p53. Consistent with the later, we found no difference in the increase of Sall2 expression in response to doxorubicin between p53-deficient (EtOH) and p53-active (4-OHT) MEFs (Figure 2e). The known p53 targets p21^WAF^ and BAX substantially increased after doxorubicin treatment in MEFs incubated with 4-OHT; however, consistent with being reported as Sall2 targets,^12, 20^ the increase of p21^WAF^ and BAX were almost lost in *Sall2*^*−/−*^ MEFs, even under the activation of p53 by 4-OHT and doxorubicin (Figure 2d). These experiments indicate that Sall2 is required for p21^WAF^- and BAX-induced expression.

### Sall2 is required for Noxa expression under genotoxic stress, and activates *NOXA* promoter

**Figure 3:**
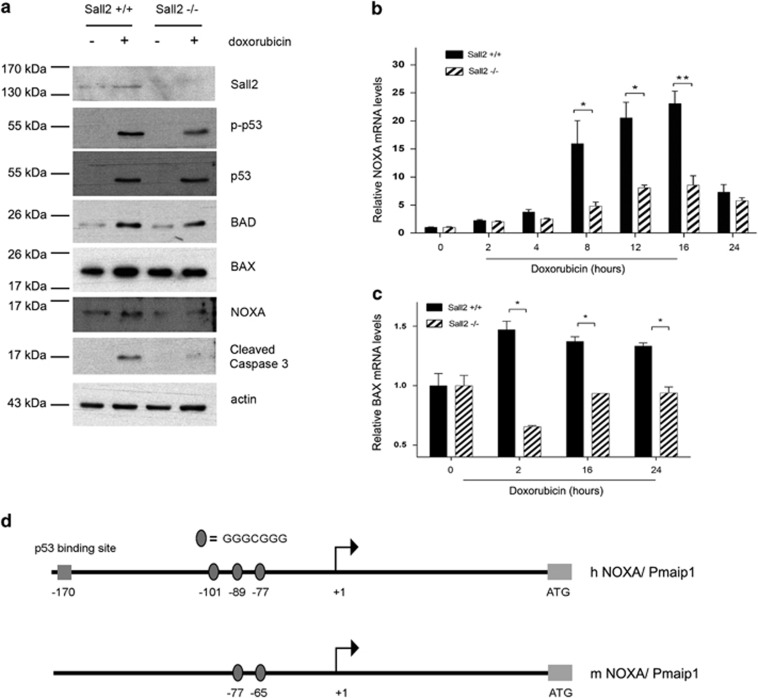

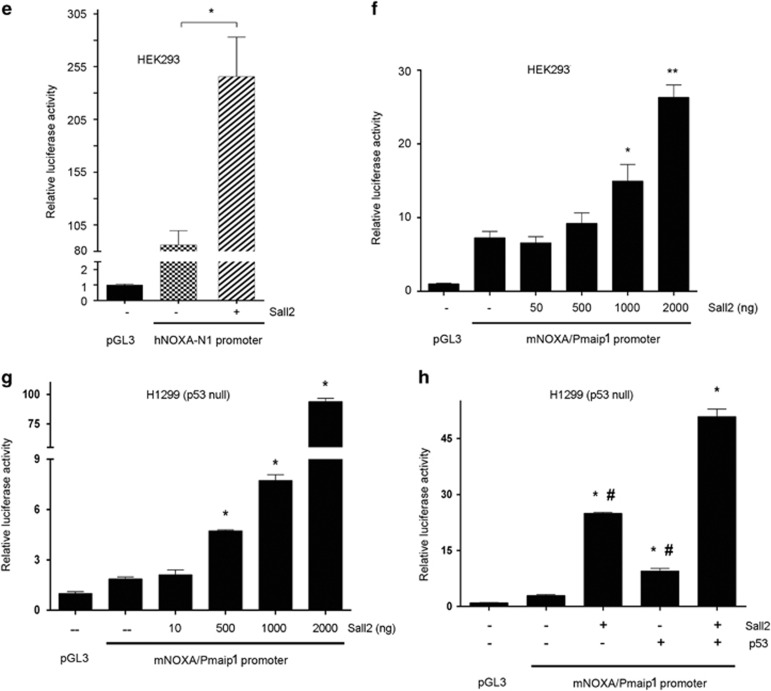
Sall2 expression correlates with induction of apoptotic markers, and increases *NOXA* promoter activity. (**a**) MEFs *Sall2*^*+/+*^ and *Sall2*^*−/−*^ were exposed to 1 *μ*M doxorubicin or vehicle (DMSO) for 16 h. Levels of Sall2, p-p53 (Ser18), p53, BAD, BAX, NOXA and cleaved caspase-3 were evaluated by western blot. Actin shows equal loading. The figure is representative of three independent experiments. (**b** and **c**) MEFs *Sall2*^*+/+*^ and *Sall2*^*−/−*^ were exposed to 1 *μ*M doxorubicin for various times. *Noxa* (**a**) and *BAX* (**b**) mRNA levels were measured by quantitative real-time PCR and is relative to *cyclophilin A.* Level at time 0 was defined as 1. Average values from triplicate samples are shown as representative of three independent experiments. *P<0.05; **P<0.005. (**d**) Schematic representation of human (h) and mouse (m) *Noxa* promoters and the location of identified Sall2 putative binding sites (represented by ovals) relative to the transcription start site (+1). (**e**) Transient co-transfection of the hNOXA-N1 promoter without and with Sall2 vector in HEK293 cells was performed as described in the Materials and Methods section. (**f**) Transient co-transfection of the mouse *Noxa* promoter without or with increasing concentrations of Sall2 vector in HEK293 cells. (**g**) Transient co-transfection of the mouse *Noxa* promoter without and with increasing concentrations of Sall2 vector in human H1299 (*p53-null*) cells. (**h**) Transient co-transfection of the mouse *Noxa* promoter with Sall2, p53 or both, in human H1299 (*p53-null*) cells as described in the Materials and Methods section. From (**e**) to (**h**), cell extracts were analyzed for luciferase activity and normalized to *β*-galactosidase activity. Promoter activity is expressed as relative luciferase activity to pGL3 empty vector. Results represent three independent experiments performed in triplicate. Error bars represent the mean±S.D. Statistical significance was determined by Student's *t*-test (*P<0.05; **P<0.001). For figure (**h**), *P<0.05 relative to pGL3 alone and ^#^*P*<0.05 relative to Sall2 and p53 together

We further investigated the apoptotic response to doxorubicin by analyzing the expression of other proapoptotic proteins. In addition to BAX, we analyzed the expression of BAD (BCL2-associated death promoter) and Noxa. In parallel, we analyzed the levels of total and phosphorylated p53 (Ser18). As indicated previously, Figure 3a shows that p53 levels and activity increase in response to doxorubicin in both *Sall2*^*+/+*^ and *Sall2*^*−/−*^ MEFs. BAD protein levels also increased in response to doxorubicin; however, the induction of BAX and Noxa were decreased in the absence of Sall2. Consistent with previous experiments, the levels of cleaved caspase-3 also decreased in *Sall2*^*−/−*^ MEFs. *BAX* has been previously reported as a Sall2 target gene,^20^ but the observed correlation between Noxa and Sall2 expression is novel.

To confirm a Sall2-dependent transcriptional regulation of *Noxa* under genotoxic stress in MEFs, we analyzed *Noxa* mRNA levels by qPCR during different times of doxorubicin treatment, and compared them between *Sall2*^*+/+*^ and *Sall2*^*−/−*^ MEFs. After 8 h of treatment, *Noxa* mRNA significantly increased in *Sall2*^*+/+*^ MEFs and continued increasing up to 16 h. In contrast, *Noxa* mRNA levels were modestly increased in *Sall2*^*−/−*^ MEFs (Figure 3b). The difference of *Noxa* mRNA induction between *Sall2*^*+/+*^ and *Sall2*^*−/−*^ MEFs was highly significant and was even more pronounced compared with that of *BAX* mRNA (Figure 3c). These results confirm that there is a Sall2-dependent regulation of Noxa and BAX during doxorubicin treatment.

A consensus sequence, GGG (T/C) GGG, for optimal binding of Sall2 was identified previously.^20^ We searched for putative Sall2-binding sites in several apoptotic-related genes including members of the BH3-only, the proapoptotic BAX-like and the antiapoptotic Bcl-2 protein subfamilies (Supplementary Figure 2). Based on the number of Sall2 putative sites present in the promoter, the proximity between the sites, the conservation between human and mouse promoter regions and the correlation between Noxa and Sall2 expression (Figures 3a and b), we investigated *Noxa*. Bioinformatic analysis identified three putative Sall2-binding sites in the human *NOXA/PMAIP1* (phorbol-12-myristate-13-acetate-induced protein 1) gene (ID 5366) at positions −77, −89 and −101 upstream of the transcription start site (represented as ovals in Figure 3d) (for latter studies named as h1, h2 and h3, respectively). Two putative Sall2-binding sites were also identified in the mouse *Noxa/Pmaip1* gene (ID 58801) at positions −65 and −77 of start site (for latter studies named as m1 and m2, respectively). Responsiveness of human *NOXA/PMAIP1* promoter −198/+45 region to Sall2 was studied using a reporter described previously.^43^ HEK293 cells were co-transfected with pGL3-NOXA-N1 reporter and the mouse Sall2 isoform E1A (Sall2-GFP), or empty vector. Figure 3e shows that expression of Sall2 significantly increased *NOXA* promoter activity. In addition to the human promoter, we cloned the −970/+325 region of mouse promoter to test its regulation by Sall2. Similarly, we observed an increase in *Noxa* promoter activity in response to increasing concentrations of Sall2 protein, with a significant increase when using 1 and 2 *μ*g of Sall2 vector (Figure 3f). Nuclear localization and concentration-dependent increase of exogenous Sall2 expression were confirmed by immunofluorescence microscopy (Supplementary Figure 3).

We also evaluated whether Sall2 mediates *Noxa* transcriptional activity independently of p53 using human cancer cells that lack p53. We co-transfected the mouse *Noxa* promoter reporter with increasing concentrations of Sall2 in H1299 (*p53-null*) lung cancer cells. Figure 3g shows that Sall2, in the absence of p53, also increased *Noxa* promoter activity in a concentration-dependent manner. Transfection of wild-type p53 increased *Noxa* promoter activity threefold compared with the eightfold increase by Sall2 (Figure 3h). When Sall2 and p53 were added together, they increased *Noxa* promoter activity more than 16-fold. Similar results were obtained by using HCT116 (*p53-null*) colon cancer cells (Supplementary Figure 4). Taken together, our data suggest that the increase on Sall2 expression positively regulates human and mouse *Noxa* gene expression, an effect that occurs in a p53-independent manner. However, similar to the regulation of the *p21*^*WAF*^ promoter,^12^ our data also suggest that Sall2 and p53 synergizes to induce Noxa expression.

### Sall2 binds to sequences located in the proximal promoter of the *Noxa* gene

**Figure 4:**
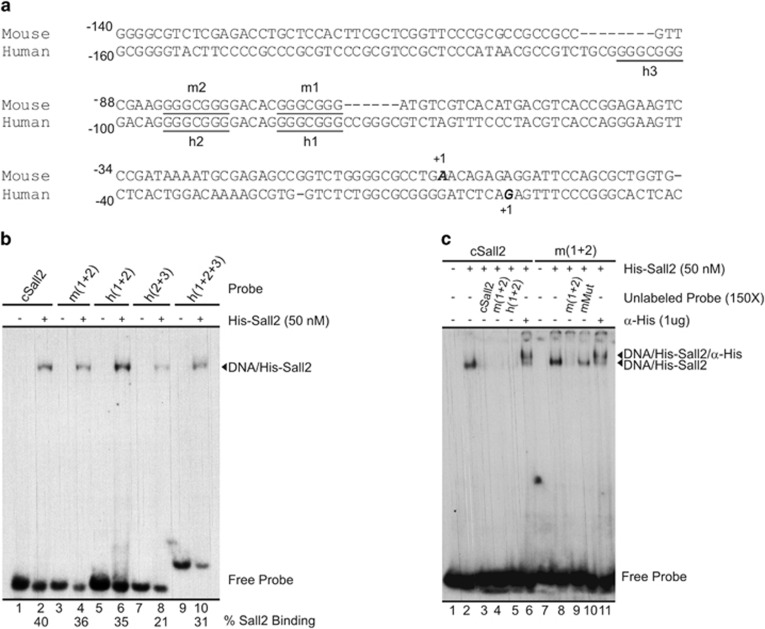
Analysis of Sall2-binding sites located at the *Noxa* gene in mouse and human promoters. (**a**) Sequence alignment of mouse and human *Noxa* promoter obtained from ClustalW. Underlined letters show Sall2-binding sites in both promoters, the putative sites are named h1, h2 and h3 (for human gene) or m1 and m2 (for mouse gene), and numbers denote the sites position from transcription start site. (**b**) EMSA assay testing double-stranded oligonucleotide probes containing the putative Sall2-binding sites located at mouse (m1+2) and human (h1+2, h2+3 and h1+2+3) promoter region of *NOXA/PMAIP1* gene, compared with consensus Sall2 double-binding site (cSall2), using recombinant His-Sall2 (50 nM). The percentage of His-Sall2 binding is depicted at the bottom of the figure. The migration of free probe and the DNA/Sall2 complex are indicated at the right side of the figure. (**c**) The cSall2 and m (1+2) probes were used in a competition and supershift analysis with His-Sall2 (50 nM). The competition was made in the presence of a 150 × molar excess of the different unlabeled normal and mutant probe, as indicated at the top of the figure. The specificity of DNA/His-Sall2 complex is corroborated by supershift using 1 *μ*g of His antibody. The migration of free probe, DNA/Sall2 and DNA/Sall2/*α*-His complexes is indicated at the right side of the figure

Figure 4a shows a sequence alignment of human and mouse *Noxa* proximal promoters, highlighting the putative Sall2 cognate sequences identified in our search (h1, h2, h3, m1 and m2). The spacing between sites 1 and 2 in the human and mouse promoters is the same (5 bp), and these sites are at a similar distance from the transcription start site in both species. We tested the ability of Sall2 to bind these sites using electrophoretic mobility shift assays (EMSA). We first used recombinant His-Sall2, expressed in *Escherichia coli* and then affinity purified (Supplementary Figures 5A and B). We analyzed the binding of His-Sall2 to four different double-stranded oligonucleotide probes containing two or three putative Sall2-binding sites (Supplementary Data 1). As a control, we used a probe containing two copies of the Sall2-binding sequence described by Gu *et al.*^20^ Sall2 binding to a probe containing two recognition sites was significantly stronger compared with a probe containing only one site (Supplementary Figure 5C). Single retardation bands were obtained by incubating any of the oligonucleotide probes with His-Sall2 (Figure 4b). Similar binding strengths were obtained when comparing the control probe to the probes containing sites 1 and 2 of the human or mouse *NOXA* gene promoter (Figure 4b, compare lane 2 to lanes 4 and 6). A weaker affinity of His-Sall2 to probes harboring sites 2 and 3 of the human *NOXA* promoter was observed (Figure 4b, compare lanes 2, 4 and 6 to lane 8). The three Sall2-binding sites found in the proximal promoter of the human *NOXA* gene display the same core sequence (GGGCGGG). This fact suggests that the weaker affinity of Sall2 to probes containing site h3 relies on DNA sequences surrounding these Sall2-binding sites. His-Sall2 binds specifically to the sites contained in the probes tested, as demonstrated by competition analyses (Figure 4c, lanes 3, 4, 5, 9 and 10). Additionally, a supershift band was obtained when using an anti-His antibody, confirming that the retardation bands were generated by His-Sall2 (Figure 4c, lanes 6 and 11).

**Figure 5:**
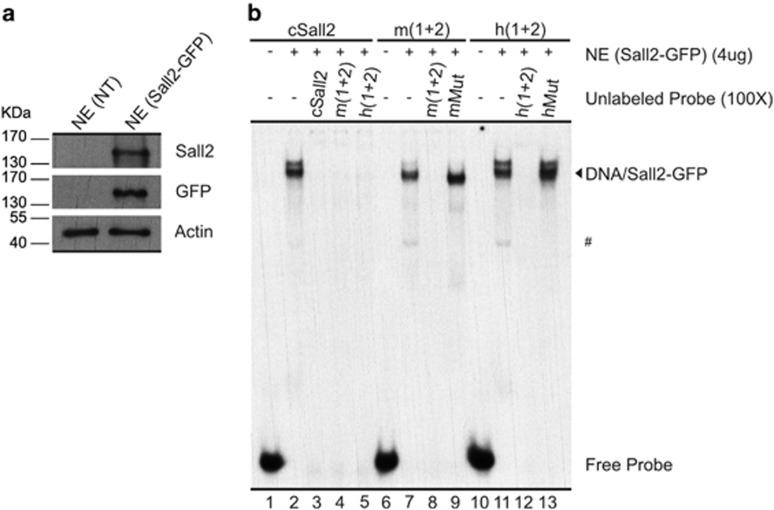
Sall2 specifically binds to a DNA sequence at the *Noxa* promoter. (**a**) Western blot analysis of nuclear extracts from HEK293 non-transfected (NT), or transfected with Sall2-GFP. Sall2 expression was confirmed using Sall2-specific (ATLAS) antibody, and anti-GFP antibody that recognizes Sall2-GFP fusion protein. *β*-Actin shows equal loading in each case. (**b**) The cSall2 and m (1+2), as well as h (1+2) probes, were used in a competition analysis using 4 *μ*g of nuclear extracts (NE, Sall2-GFP) in the presence of a 100 × molar excess of the different unlabeled normal and mutant probe (three point mutations in each binding site), as indicated at the top of the figure. The competition was performed in the presence of a 100 × molar excess of the different unlabeled normal and mutant probe, as indicated at the top of the figure. The migration of free probe and DNA/Sall2-GFP complexes is indicated at the right side of the figure. ^#^Corresponds to a faster migrating band (see comments in the text)

Subsequently, we performed EMSA analyses using nuclear extracts obtained from HEK293 cells transfected with a vector coding for Sall2-GFP. The expression of Sall2 was assessed by western blot analyses (Figure 5a). Non-detectable levels of endogenous Sall2 were observed when using an anti-Sall2 antibody (Figure 5a; no transfection: NT). We observed a strong binding to the probes harboring the mouse or human *NOXA* promoter, sites 1 and 2 (Figure 5b, lanes 7 and 11). The retardation band is generated by Sall2-GFP in the nuclear extracts as a supershift is observed when adding an anti-GFP antibody (Supplementary Figure 6A), and reactions using nuclear extracts from non-transfected cells did not result in the generation of these bands (Supplementary Figure 6B, lanes 2, 5 and 8). Competition analyses confirmed the specificity of this binding (Figure 5b, lanes 8, 9, 12 and 13). Minor faster and slower migrating bands were also observed using the nuclear extracts containing Sall2-GFP (Figure 5b, lanes 2, 7 and 11). The faster migrating bands were also observed when using nuclear extracts from non-transfected cells (Supplementary Figure 6B), suggesting the presence of other proteins with affinity for GC-rich sequences in these cells. The slower migrating band appears only with the use of Sall2-GFP-containing nuclear extracts. It might reflect Sall2-GFP interaction with other nuclear proteins, the presence of posttranslational modifications in a subset of Sall2-GFP or interaction of an additional unit of Sall2-GFP. Taken together, our EMSA analyses indicate that the stimulatory effect of Sall2 in the reporter assays corresponds to a direct effect exerted by this transcription factor.

### Sall2 interacts *in vivo* with a specific region of *NOXA* promoter

**Figure 6:**
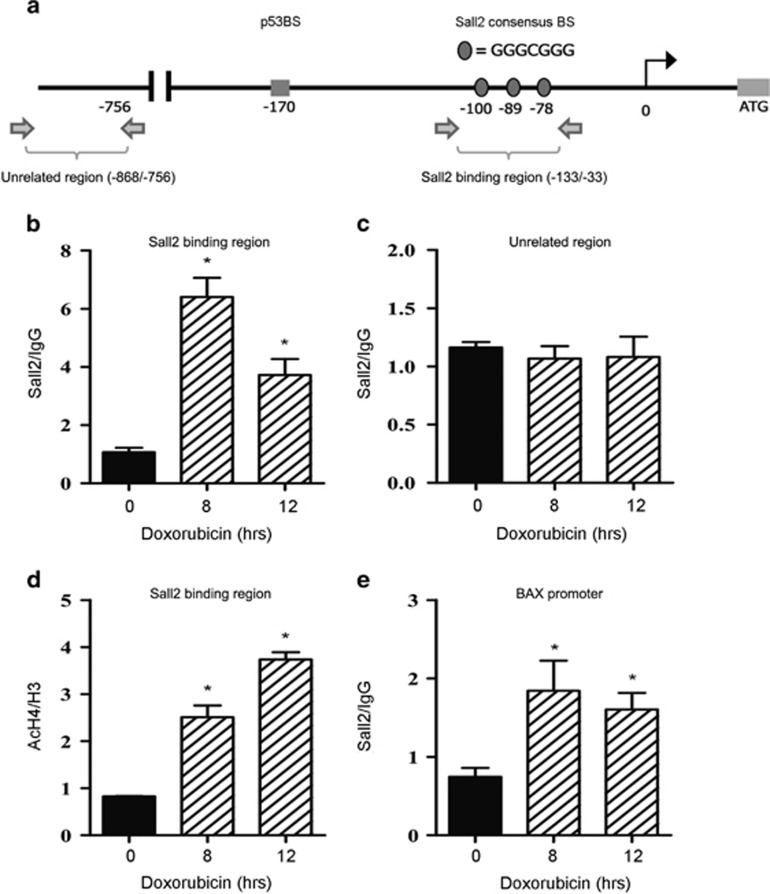
Sall2 interacts with *NOXA* promoter *in vivo*. (**a**) Schematic representation of the human *NOXA* promoter. Horizontal arrows indicate the location of primers used for qPCR in site-specific ChIP assays. Note that the figure is not drawn to scale. Black arrow: transcription start site. (**b**, **c** and **e**) HEK293 cells were treated with doxorubicin for 8 and 12 h and then anti-Sall2 antibody (Bethyl Lab) or normal rabbit IgG (control antibody) was used for ChIP. Real-time PCR was performed using primers that amplify the putative Sall2 region (−133/−33) (**b**), a nonspecific negative control region (−868/−756) where no binding is expected to occur (**c**) and the proximal region of the *BAX* promoter used as a positive control for Sall2 binding^20^ (**d**). Changes in acetylation levels on nucleosomes located in the (−133/−33) region of the human *NOXA* promoter were measured by the ratio of acetylated H4(AcH4) over total histone content (H3). Graphs in (**b**, **c** and **e**) show quantification of the amplified DNA for each immunoprecipitation relative to IgG. Error bars represent 1 S.D. for PCR reactions performed in triplicate from representative ChIP assay. Statistical significance was determined by Student's *t*-test (*P<0.05). BS, binding site

To demonstrate *in vivo* the interaction of Sall2 with *NOXA* promoter, we performed chromatin immunoprecipitation (ChIP) assays on HEK293 cells using a recently characterized Sall2 antibody.^4^ Cells were treated with doxorubicin for various times, chromatin was immunoprecipitated and specific genomic regions were analyzed by qPCR. Figure 6a shows a representation of the *NOXA* promoter and the chromatin regions evaluated by amplification. Consistent with all our previous data, Sall2 significantly increased *in vivo* its binding to the *Noxa* promoter region containing the Sall2-specific binding sites during doxorubicin treatment (Figure 6b). In contrast, no binding of Sall2 was observed to a promoter region upstream of the Sall2-specific binding sites (Figure 6c). The increment of Sall2 binding was correlated with an increase in histone H4 acetylation, a transcriptional activation marker (Figure 6d). Of note, an effective binding after doxorubicin treatment was found for a previously defined region of the *BAX* promoter,^20^ which served as a positive control (Figure 6e). However, Sall2 binding to *BAX* promoter was less pronounced compared with that observed for the *Noxa* promoter (2.0- *versus* 6.0-fold enhancement, respectively).

### Doxorubicin-dependent Sall2/Noxa axis in Jurkat cells

**Figure 7:**
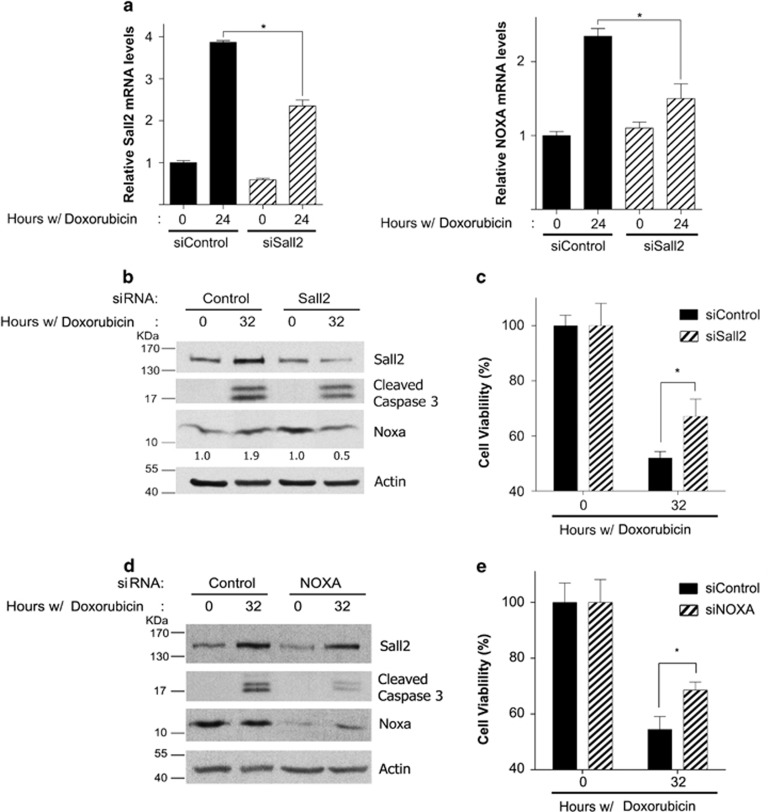
Doxorubicin-dependent Sall2/Noxa axis in Jurkat cells. Jurkat cells were transiently transfected with control or Sall2-specific small interfering RNA (siRNA). Cells were then treated with 0.5 *μ*M doxorubicin for 24 and 32 h to evaluate mRNA, proteins and cell survival. Sall2 (**a**, left) and Noxa (**a**, right) mRNA levels were evaluated after 24 h. Doxorubicin treatment using quantitative real-time PCR. Expression of Sall2 and Noxa are relative to *cyclophilin A*, the expression at time 0 for siControl was defined as 1. Values from triplicate samples are representative of two independent experiments. Each bar represents the mean±S.D. Statistical significance was determined by Student's *t*-test (*P<0.05). (**b**) Cell lysates were collected after 32 h to evaluate Sall2, cleaved caspase-3 and Noxa proteins by western blot. The ratio of Noxa to actin was measured by densitometry and the fold increase of Noxa over that at time 0 was calculated (values are shown below Noxa western blot). Figure is representative of two independent experiments. (**c**) Cellular viability was measured after 32 h doxorubicin treatment using a CytoTox-Glo kit assay. The percentage (%) of cell viability is plotted as the mean±S.D. of two independent experiments performed in triplicate. The percentage at time 0 for each siRNA was defined as 100%. Statistical significance was determined by Student's *t*-test (*P<0.05). (**d**) Jurkat cells were transiently transfected with Control or Noxa siRNA and treated with doxorubicin as in (**b**). Cell lysates were collected and analyzed by western blot. Actin shows equal loading. (**e**) Jurkat cells were transiently transfected with Control or Noxa siRNA and treated with doxorubicin as in (**b**), and then cellular viability was measured as in (**c**). The percentage (%) of cell viability is plotted as the mean±S.D. of two independent experiments performed in triplicate. The percentage at time 0 for each siRNA was defined as 100%. Statistical significance was determined by student *t*-test (*P<0.05)

To demonstrate the relevance of the Sall2/Noxa axis in a human cancer cell model, we used Jurkat leukemia T cell. Because these cells are null for p53 and BAX,^44, 45, 46^ they allow us to investigate the role of Sall2 in cell death, in a p53- and BAX-independent manner. In addition, it has previously been shown that in Jurkat cells Noxa is essential for genotoxic agent etoposide-^46^ and proteasome inhibitor bortezomib-^47, 48^ induced apoptosis, both drugs relevant for cancer treatments. Jurkat cells were treated with control or Sall2-specific siRNA and then exposed to doxorubicin for 24 and 32 h. We used the 24- h time point for qPCR analysis to determine *SALL2* and *NOXA* mRNA levels in response to doxorubicin, and the 32-h time point for western blot and functional assays. Figure 7a shows that *SALL2* and *NOXA* mRNAs increased in response to doxorubicin (3.8- and 2.3-fold, respectively). Sall2 depletion by siRNA (Figure 7a, left panel) resulted in a significant decrease in *NOXA* mRNA induction (Figure 7a, right panel). Protein analysis confirmed the induction of Sall2 and Noxa (Figure 7b). However, the increase of Noxa protein was modest (1.9-fold) probably because of constitutive Noxa expression as reported previously,^49^ and/or a decrease in protein stability because incubating cells with a proteasome inhibitor, MG132 (carbobenzoxy-Leu-Leu-leucinal), increases Noxa protein levels (Supplementary Figure 7). We were unable to observe any increase in Noxa protein in response to doxorubicin in Sall2 siRNA-treated cells, which was correlated with a decrease in the levels of cleaved caspase-3 (Figure 7b). As the latter result suggested a decrease in the apoptotic response, we also evaluated Sall2-dependent cell survival. Figure 7c shows that doxorubicin decreased cell survival by 50%, and Sall2 depletion partially, but significantly, reverted this effect. Finally, to confirm that Noxa is involved in the cell death response in this cancer cell model, cells were transfected with control or Noxa-specific siRNA and then treated with doxorubicin as above. Figure 7d shows that Noxa depletion decreased the levels of cleaved caspase-3, which correlated with a significant increase on cell viability (Figure 7e). These results altogether indicate that in a p53- and BAX-independent context Sall2 is needed for Noxa induction, a necessary step for full cell death response to doxorubicin in Jurkat cells.

## Discussion

Increasing evidences indicate that alterations in the function of Sall2 have a role in disease, including cancer, ocular coloboma and kidney dysfunction.^1, 2, 3, 4^ How Sall2 is involved in cancer is still controversial. Evidence have supported a role for Sall2 as a tumor suppressor.^3, 12, 13, 50^ However, Sall2 is upregulated in various human cancers^15, 16, 17, 18, 19, 51^ and is essential for glioblastoma propagation.^4^ These evidences suggest that the role of Sall2 is cell context-dependent. Thus, identification of Sall2 function and targets under different cell context are essential to understand its role in disease. We have recently demonstrated that p53 regulates Sall2 under genotoxic stress.^27^ Although the study could not provide a functional explanation for that regulation, here we demonstrated that Sall2 has an essential role in the apoptotic response to extended genotoxic stress. In support of this role, we identified the proapoptotic *NOXA* gene as a novel Sall2 target.

Our previous report suggested that Sall2 and p53 have opposite roles under genotoxic stress because Sall2 is negatively regulated by p53 under this condition. Here, analysis of Sall2 expression over the period of doxorubicin treatment demonstrated that Sall2 returns and increases after extended treatment, and localizes exclusively in the nucleus. Our results suggest that Sall2 and p53 rather cooperate in the apoptotic response. However, we also showed that the increase on Sall2 is independent of p53, and that Sall2 is relevant for apoptosis even in the absence of p53. Why Sall2 is downregulated at early times of genotoxic stress is unknown. p53 might initially downregulate Sall2 to support DNA repair and survival, but Sall2 is then necessary to cooperate with p53 to promote apoptosis if irreversible DNA damage occurred. In agreement, we found that Sall2 and p53 cooperate to activate the *NOXA* promoter. Similarly, independent and additive effects of Sall2 and p53 on the *p21*^*WAF*^ promoter have been reported, suggesting that Sall2 and p53 potentiate each other functions under certain cellular contexts. Consequently, loss of p53 and murine Sall2 synergistically promotes lymphomagenesis.^13^ *Sall2*^*−/−*^ or *Sall2^−/+^; p53*^*−/−*^ mice, compared with the *Sall2^+/+^;p53*^*−/−*^ mice, exhibit accelerated tumorigenesis and advanced tumor progression.^13^ All these results prompt further investigation to understand the regulatory and functional relationship between Sall2 and p53.

A role for Sall2 in apoptosis is in agreement with previous reports. Overexpression of Sall2 inhibits DNA synthesis and increases apoptosis of ovarian cancer cells, effects accompanied by increased expression of p21^WAF1^ and BAX proteins.^12^ Consistently, Sall2 was found to directly regulate the proapoptotic *BAX* after treatment of human ovarian surface epithelial (HOSE) cells with etoposide.^20^ In addition, treatment of HOSE cells with etoposide slightly increases both Sall2 levels and Sall2 binding to the *c-Myc* promoter.^21^ The latter was associated with *c-Myc* repression and cellular apoptosis. However, Sall2 could have a prosurvival role during normal brain development.^8^ All these studies are consistent with a cell-context-dependent function for Sall2. Our results, in mouse embryonic fibroblast, reinforce *BAX* as a conserved Sall2-dependent gene induced under genotoxic stress, and support that Sall2 is a stress-responsive molecule that promotes apoptosis under genotoxic stress.

Previous studies identified a consensus sequence for optimal binding of Sall2 *in vitro*, the sequence GGG (T/C) GGG was identified in human *BAX* and *c-Myc* gene promoters and the binding of Sall2 to these promoters was confirmed.^20^ Our studies confirmed that *Noxa* is a novel target for Sall2. Consequently, mRNA and protein analysis revealed that doxorubicin-dependent induction of Noxa was decreased in *Sall2* ^*−/−*^ MEFs, whereas induction of proapoptotic BAD was not affected by the lack of Sall2, suggesting a specific transcriptional regulation of apoptotic-related genes during genotoxic stress. The Sall2-dependent doxorubicin-induced Noxa expression was also confirmed in Jurkat T leukemia cells, suggesting that the Sall2/Noxa axis happens in a cancer cell context.

Noxa is a central mediator of stress responses and critical for setting the apoptotic entrance.^28, 52^ Noxa shows weak proapoptotic potential on its own, but is critical in fine-tuning cell death decisions because it targets for degradation Mcl-1, a prosurvival protein.^53, 54^ *Noxa* was initially defined as a p53-inducible gene in response to cellular stress.^55, 56^ However, depending on the cell type and/or extension of a specific stress, *Noxa* can also be induced independently of p53 by other transcription factors, including p73, E2F1, HIF-1*α*, c-Myc, CREB and myocardin-related transcription factor A.^36, 48, 57, 58, 59, 60^ In response to etoposide- or doxorubicin-induced DNA damage in MEFs, Noxa expression was shown to be strictly dependent on p53.^36^ This observation is apparently in disagreement with our observation that Sall2 is required for an efficient induction of Noxa by doxorubicin. A plausible explanation is that previous studies were carried out in a normal Sall2 context. Thus, together with our results, they indicate that Sall2 and p53 are required for the induction of Noxa. On the other hand, our studies in a cancer cell model, wherein p53 and BAX are absent, suggest that Sall2 is relevant for Noxa expression, and for the cell death response under genotoxic stress. However, we cannot conclude that only Noxa is responsible for the Sall2-dependent response to doxorubicin. Other factors, not yet identified, could be transcriptionally regulated by Sall2 and contribute to the apoptotic response. Studies in HOSE cells showed that Sall2 represses MYC under etoposide treatment, or when Sall2 is overexpressed.^21^ On the other hand, MYC is an activator of the *NOXA* gene upon proteasome inhibitor treatment in melanoma and HeLa cells.^48^ The relationship between c-Myc, Sall2, and Noxa is not straightforward because studies are from different cell types and context. Myc has both proliferation and apoptotic activities; these activities are context- and cell-dependent, but also threshold-dependent.^61^ Similarly, the Sall2 actions might depend on the cellular context, type of stress or threshold levels. To get a direct relationship, c-Myc, Sall2 and Noxa should be looked at in the same cell type, as the effect might be different from one cell to another. Further studies are needed to understand whether the requirement of Sall2 for the expression of Noxa and/or for the apoptotic response is tissue-specific, or dependent on a specific stimulus within the same cell type, and how it relates with the levels of other transcription factors that could also regulate Noxa.

As most compounds used to treat cancer induce apoptosis, factors that influence apoptosis may contribute to the outcome of cancer therapy. Our study highlights the role of Sall2 in genotoxic stress-dependent apoptosis and the identification of the proapoptotic *NOXA* gene as a novel Sall2 target. We predict that future studies on the functional relationship between Sall2 and p53 will provide new avenues through our understanding of normal Sall2 function as well as its role during disease and treatments.

## Materials and Methods

### Reagents

Doxorubicin, 4-OHT, MG132, anti-actin and anti-Sall2 (HPA004162) antibodies were purchased from Sigma (St. Louis, MO, USA). Normal rabbit IgG, p21 monoclonal (H5) and GFP (B-2) antibodies were obtained from Santa Cruz Biotechnology (Santa Cruz, CA, USA). Anti-Bad (no. 9292), anti-Bax (no. 2772), anti-cleaved PARP (Asp214) (no. 9544), anti-cleaved caspase-3 (Asp175) (no. 9661) and anti-phosphorylated p53-Ser15 (no. 9284) were purchased from Cell Signaling Technology (Danvers, MA, USA). Anti-Sall2 antibody (no. A303-208) used for ChIP experiments was obtained from Bethyl Lab (Montgomery, TX, USA). 6 × His monoclonal antibody (no. 631212) was obtained from Clontech (Mountain View, CA, USA). Anti-Noxa (no. 13654) was purchased from Abcam (Cambridge, UK). Anti-p53 (Pantropic PAb421; no. OP03) was obtained from Calbiochem (Billerica, MA, USA). Anti-histone H3 and anti-histone H4 acetylated were obtained from Millipore (Billerica, MA, USA). Control (no. 37007) and NOXA (no. 37305) siRNAs were obtained from Santa Cruz Biotechnology, and Sall2 (no. AM16708A) siRNA was from Ambion-Life Technologies Inc., Delegación Cuauhtémoc, Mexico DF, Mexico.

### Plasmids

The human *NOXA*/*PMAIP1* promoter (a gift from Yihong Ye, NIDDK, Bethesda, MD, USA) was described previously^62^ and was obtained from Addgene (Cambridge, MA, USA; plasmid no. 26112). The mouse *Noxa/Pmaip1* promoter was cloned from genomic DNA from a wild-type mouse, using the following oligonucleotides: forward, 5′-GTACAGATCTTTCACTTCAGAAGGGCGTTGCT-3′ and reverse, 5′-AAACAAGCTTACATGCAGGCGCGTACATTCTA-3′, and then the purified promoter was subcloned into the pGL3-Basic plasmid. The coding sequence for full-length mouse *Sall2* was synthetized by GeneScrip (http://www.genscript.com/) according to the Sall2 codifying sequence published in the Sanger database (http://www.sanger.ac.u) and was designed in fusion with the C-terminal GFP tag. Mouse *Sall2* sequence was then subcloned into pCDNA3 and pQE-80 L vectors. Fidelity of the mouse promoter and full-length *Sall2* coding sequences were confirmed by sequencing analysis at Pontificia Universidad Católica Sequence Facility, Santiago, Chile. The pCMV-Neo-Bam p53 wild-type plasmid was a gift from Bert Vogelstein (Addgene; plasmid no. 16434).^63^

### Cell culture

HEK293 human kidney epithelial cells (ATCC, Manassas, VA, USA; CRL-1573), HCT116 (*p53-null*) human colon cancer cells (a gift from Dr. Robert Warren, University of California San Francisco, San Francisco, CA, USA), *Sall2* ^*+/+*^, *Sall2* ^*−/−*^ and *p53*^*ER/ER*^ MEFs were cultured in Dulbecco's modified Eagle's medium (DMEM) (Hyclone, Logan, UT, USA) supplemented with 10% (v/v) fetal bovine serum (FBS; Hyclone), 1% glutamine (Invitrogen Santa Fe, Mexico DF, Mexico) and 1% penicillin/streptomycin (Invitrogen). H1299 (*p53-null*) human lung cancer cells (ATCC; CRL-5803) and Jurkat leukemia cells (a gift from Dr. Giancarlo de Ferrari, University Andres Bello, Santiago, Chile) were cultured in RPMI (Roswell Park Memorial Institute Medium) (Hyclone) supplemented with 10% (v/v) FBS, 1% glutamine and 1% penicillin/streptomycin. Experiments with *p53*^*ER/ER*^ MEFs and *Sall2*^*−/−*^ MEFs were performed with early passages (before passage 4). When indicated, it was added to the medium containing 100 nM of 4-OHT (Sigma) in 100% EtOH, or an equal volume of EtOH control. For genotoxic stress and p53 activation, doxorubicin was added to the cell culture at indicated concentrations and times (see figure legends).

### Isolation of primary MEFs and genotyping

*Sall2* knockout mice^9^ were obtained by collaboration with Dr. Ruichi Nishinakamura (Kumamoto University, Kumamoto, Japan; MTA (2010) to RP, Universidad de Concepción, Concepción, Chile). Similarly, the *p53*^*ER/ER*^ mice^29^ were obtained by a collaborative work with Dr. Gerard Evan (University of California San Francisco and University of Cambridge, Cambridge, UK). *Sall2*^*+/−*^ mice were crossed to generate isogenic *Sall2*^*+/+*^ and *Sall2*^*−/−*^ embryos. Mice were group housed under standard conditions with food and water available *ad libitum*, and were maintained on a 12 h light/dark cycle. Mice were fed a standard chow diet (Lab Diet, St Louis, MO, USA) containing no <5% crude fat and were treated in compliance with the US National Institutes of Health guidelines for animal care and use. Studies were reviewed and approved by the Animal Ethics Committee of the Chile's National Commission for Scientific and Technological Research (CONICYT, protocol for project no. 1110821).

Fibroblasts from *Sall2*^*+/+*^, *Sall2*^*−/−*^, *Sall2*^*+/+*^*; p53*^*ER/ER*^ and *Sall2*^*−/−*^*; p53*^*ER/ER*^ were prepared from embryos at 13.5 days *post coitum* as described previously.^9^ Briefly, embryos, whose head and other red organs were removed, were smashed into pieces using a razor blade in a 10-cm dish with 5 ml trypsin (Hyclone). The smashed embryo was incubated in trypsin for 15 min at 37 °C followed by dilution in 10 ml DMEM by pipetting up and down. The cells were centrifuged and seeded in 100-mm culture dishes (passage 0). MEFs were generated from independent embryos and routinely cultured as described above.

Mice were routinely genotyped by isolating tail DNA as reported previously.^27^ One microliter of genomic DNA was used for PCR analysis. *Sall2* PCR was performed as described previously^9^ with the following oligonucleotides: forward, 5′-CACATTTCGTGGGCTACAAG-3′ and reverse, 5′-CTCAGAGCTGTTTTCCTGGG-3′ and Neo, 5′-GCGTTGGCTACCCGTGATAT-3′. The sizes of the PCR products are 188 bp for the wild-type mutant and 380 bp for the null mutant. For the *p53*^*ER/ER*^ model,^28^ p53 genotyping^29^ was carried out with the following oligonucleotides: forward, 5′-CCTCCAGCCTAGAGCCTTCCAAGC-3′ and reverse, 5′-GGTGAGATTTCATTGTAGGTGCC-3′ and Neo, 5′-GCACACAAACTCTTCACCCTGC-3′. The sizes of the PCR products are 430 bp for the wild-type mutant and 700 bp for the null mutant. All PCRs were performed for 32 cycles with annealing temperature for Sall2 at 58 °C and for p53 at 66 °C.

### Western blot analysis

Proteins from cell lysates (50–70 *μ*g of total protein) were fractionated by SDS-PAGE and transferred for 1 h at 200 mA to PVDF membrane (Immobilon; Millipore) using a wet transfer apparatus. The PVDF membranes were blocked for 2 h at room temperature in 5% nonfat milk in TBS-T (TBS with 0.1% Tween), and incubated with primary antibody at an appropriate dilution at 4 °C overnight in blocking buffer. After washing, the membranes were incubated with horseradish peroxidase-conjugated secondary antibodies diluted in TBS-T buffer for 30 min at room temperature. Immunolabeled proteins were visualized by ECL (Pierce, Thermo Scientific, Waltham, MA, USA).

### Transient transfections and reporter gene assays

To evaluate *Noxa* promoter transcriptional activity, HEK293, H1299 (*p53-null*) or HCT116 (*p53-null*) cells were transiently co-transfected with 0.75 *μ*g of NOXA-luc, 0.125 *μ*g of RSV-*β*-galactosidase (*β*-Gal) and various concentrations of Sall2 or control vector per well. To evaluate the effect of Sall2 and p53 in *Noxa* promoter activity in H1299 and HCT116 (*p53-null*) cells, we used 2 *μ*g of each Sall2, p53 or both, and equivalent amount of vector control for comparative analysis. After 48 h, the transfected cells were washed with phosphate-buffered saline, lysed with reporter assay lysis buffer (Promega, Madison, WI, USA) and spun at 14 000 × *g* to pellet cell debris. The supernatant was then assayed for luciferase and *β*-Gal activity using the manufacturer's suggested protocols (Promega). Luminescent reporter activity was measured using a Luminometer (Victor3; Perkin-Elmer). All transfections were normalized to *β*-Gal activity and performed in triplicate. Luciferase values were expressed as fold induction relative to the pGL3 vector control, or in some experiments to NOXA-luc. Statistical significance of X *versus* Y-treated samples was determined by one-tailed Student's *t-*test.

### Apoptosis and viability assays

For apoptosis assays in cell culture, we measured activation of caspase-3 and -7. Briefly, cells were seeded at 5 × 10^3^ cells per 96-well plates. The next day, cells were incubated with 1 *μ*M doxorubicin for 16 h and analyzed for caspase-3/7 activities using as a substrate the tetrapeptide sequence DEVD fused to aminoluciferin (caspase-Glo 3/7 assay; Promega), according to the manufacturer's instructions. After caspase cleavage, aminoluciferin is released, producing luminescence from a coupled luciferase reaction. Luminescence was measured with a microplate luminometer (Victor3; Perkin-Elmer). All assays were performed in quintuplicate. For viability assays, we used CytoTox-Glo assay (Promega), which measures dead-cell protease activity using the luminogenic peptide AAF-Glo substrate. Briefly, cells were transfected (Jurkat) or not (MEFs), and seeded at 5 × 10^3^ cells per 96-well plates. The next day, cells were incubated with doxorubicin at concentrations and times indicated in figure legends. To determine cell viability, we first measured luminescence of dead cells and then, after adding the lysis buffer, we measured luminescence of the total cells. Cell viability was calculated subtracting the luminescence of dead cells to the total cells. Luminescence was measured with a microplate luminometer (Victor3; Perkin-Elmer). All assays were performed in triplicate.

### Real-time quantitative reverse transcription-PCR

Total RNAs were extracted from cells with Trizol reagent (Life Technologies Inc.) according to the manufacturer's instructions. Before qPCR, the RNA was treated with Turbo DNase (Ambion) to eliminate any residual DNA from the preparation. One microgram of the total RNA was reverse transcribed using the Maloney murine leukemia virus reverse transcriptase (Invitrogen) and 0.25 *μ*g of Anchored Oligo(dT) 20 Primer (Invitrogen; 12577-011). qPCR was performed using KAPA SYBR FAST qPCR Master Mix Kit and the MX3000p Instrument (Stratagene, La Jolla, CA, USA) according to the manufacturer's instructions. The thermal cycling variables used were as follows: 40 cycles at 95 °C for 5 s and 60 °C for 20 s. To control specificity of the amplified product, a melting-curve analysis was carried out. No amplification of unspecific product was observed. Amplification of *cyclophilin A* was carried out for each sample as an endogenous control. Primer sequences were 5′-AGGAAGGAAGTTCCGCCG-3′ (forward) and 5′-AGCGTTTCTCTCATCACATCACA-3′ (reverse) for mouse *Noxa*, 5′-GATCTCCTCCGCAGTCTGG-3′ (forward) and 5′-ACACAATGGGTATCCGGTCT-3′ (reverse) for mouse *Sall2,* 5′ GGAGCAGCTTGGGAGCG 3′ (forward) and 5′ AAAAGGCCCCTGTCTTCATGA 3′ (reverse) for *BAX* and 5′-TTGTGGCCTTAGCTACAGGA-3′ (forward) and 5′-GCTCACCGTAGATGCTCTTT-3′ (reverse) for mouse and human *Cyclophilin A*, 5′-GCACTCGGAGACAGATGACA-3′ (forward) and 5′-CGCTTCCCCTATGTGCTAGA-3′ (reverse) for human *SALL2*, and 5′-CAGAGCTGGAAGTCGAGTGT-3′ (forward) and 5′-AGGAGTCCCCTCATGCAAGT-3′ (reverse) for human *NOXA*. The relative expression ratio of the *NOXA* and *SALL2* genes was calculated using the standard curve method, using untreated (vehicle) cells as reference. Expression of *Sall2*, *Noxa* and *BAX* were relative to *cyclophilin A.*

### Recombinant protein, nuclear extracts and EMSA

Recombinant Sall2 protein was purified as an N-terminal His-tag fusion protein using Ni-NTA agarose resin (cat. no. 30210; Qiagen, Hilden, Germany), according to the manufacturer's instructions. The protein stocks were concentrated using Amicon Ultra-4 100 kDa (Millipore). The quality of purified His-Sall2 was confirmed by Coomassie staining and by western blot using anti-His and anti-Sall2 antibodies. Nuclear extracts were obtained from HEK293 cells, transiently transfected with a vector coding for Sall2-GFP. The extracts were obtained according to the Dignam method^64^ and the presence of Sall2-GFP was confirmed by western blot. EMSAs were performed using 100 ng (50 nM) of purified His-Sall2 or 4 *μ*g of nuclear extract. Twenty femtomoles of ^32^P-end-labeled oligonucleotide probes were used separately in a 20* μ*l total binding reaction volume (see sequence information in Supplementary Data 1), including 100 ng of pBluescript DNA (*Hinf*II-digested). Binding reactions were adjusted to the following final conditions: 10 mM HEPES (pH 7.5), 100 mM KCl, 3 mM DTT, 0.05% NP-40, 3% glycerol, 5 *μ*g/ml BSA, 2.5 mM MgCl_2_ and 5 *μ*M ZnCl_2_. The reactions were incubated for 30 min at 30°C and then the samples were subjected to electrophoresis in a non-denaturing polyacrylamide gel (5% (w/v); acrylamide : bis-acrylamide ratio 60 : 1; 0.3 × TBE) at 200 V. Later, gels were dried and subjected to autoradiography. Supershift analyses were carried out by incubating His-Sall2 with a His antibody for 30 min at 4 ºC, before proceeding with the EMSA binding reaction.

### ChIP assay

ChIP assay was carried out as described previously^65^ with the following modifications: HEK293 cells were grown on 100-mm dishes to 80% confluency and then treated with 5 *μ*M of doxorubicin for 0, 8 and 12 h. Cell nuclei were sonicated to shear DNA in 300 *μ*l of sonication buffer, using a Misonix sonicator (model 3000) (18 times, 15 s on/20 s off each time, 6–9 W potency), obtaining lengths between 300 and 600 bp. Immunoprecipitations were carried out overnight at 4 °C using 5 *μ*g of Sall2 (anti-Sall2; Bethyl Lab), 1 *μ*g of H3 (anti-histone H3; Upstate), 1 *μ*g of acH4 (anti-histone H4 acetylated; Upstate) or 5 *μ*g normal rabbit IgG antibodies (Santa Cruz) and 40 *μ*g of chromatin. DNA was analyzed by real-time PCR directed to *NOXA*/*Pmaip1* promoter Sall2-specific regions (−133/− 33), and the region (−868/− 756) served as a negative control of Sall2 binding. The *BAX* promoter region (−183/+70) was used as a positive control for Sall2 binding.^20^ All the primers used are summarized in Supplementary Data 1. All PCR reactions (KAPA SYBR FAST qPCR; Kappa Biosystems, Wilmington, MA, USA) contained 1 *μ*l of input and 3 *μ*l of IP samples. Real-time PCR data were analyzed using the standard curve method.

## Abbreviations

4-OHT: 4-hydroxytamoxifen
BAX: BCL2-associated X protein
BAD: BCL2-associated death promoter
BCL2: B-cell lymphoma 2
BH3: BCL2 homology domain 3
ChIP: chromatin immunoprecipitation
DMEM: Dulbecco's Modified Eagle's Medium
DMSO: dimethyl sulfoxide
EMSA: electrophoretic mobility shift assay
ER/ER: estrogen receptor
EtOH: ethanol
FBS: fetal bovine serum
GFP: green fluorescent protein
HOSE: human ovarian surface epithelial
MEF: mouse embryo fibroblast
MG132: carbobenzoxy-Leu-Leu-leucinal
p21^WAF^: protein 21 wild-type p53 activation factor
PARP: poly (ADP-ribose) polymerase
*PMAIP1*: phorbol-12-myristate-13-acetate-induced protein 1
