# Supplementary material for: Sall2 is required for proapoptotic Noxa expression and genotoxic stress-induced apoptosis by doxorubicin
Source: Cell Death Dis. 2015 Jul 16;6(7):e1816–. doi: 10.1038/cddis.2015.165 (PMC4650718; doi:10.1038/cddis.2015.165)
Supplement: Supplementary Data 1 [file cddis2015165x1.doc]

**A**

CSall2_Dsite CGATCGGGGCGGGGCGAGGGGCGGGCCGATC

Mouse 1+2 TCGAAGGGGCGGGGACACGGGCGGGATGTCG

Human 1+2 GGACAGGGGCGGGGACAGGGGCGGGCCGGGC

Human 2+3 TCTGCGGGGCGGGGACAGGGGCGGGGACAGG

Human 1+2+3 TCTGCGGGGCGGGGACAGGGGCGGGGACAGGGGCGGGCCGGGC

Mouse Mut TCGAAGGGagaGGGACACGGagaGGATGTCG

Human Mut GGACAGGGagaGGGACAGGGagaGGCCGGGC

**B**

-132 Pmaip1 GTCCGCTCCCATAACGCC

-869 Pmaip1_NC TGAAGCGGCTCTCAGTAACC

-183 BAX GGCGCCACTGCTGGCACTTA

Pmaip1_RT GCAGAGCTGGAAGTCGAGTGT

PPIB_RT TTGTGGCGTTAGCTACAGGA

**Supplementary Data 1**. Sequences of oligonucleotides used in EMSA (**A**) and ChIP (**B**) experiments. In **A**, the core sequences of direct repeats are underlined, and the mutant nucleotides are in lowercase.
