# Supplementary material for: Sall2 is required for proapoptotic Noxa expression and genotoxic stress-induced apoptosis by doxorubicin
Source: Cell Death Dis. 2015 Jul 16;6(7):e1816–. doi: 10.1038/cddis.2015.165 (PMC4650718; doi:10.1038/cddis.2015.165)
Supplement: Supplementary Figure 1 [file cddis2015165x2.doc]

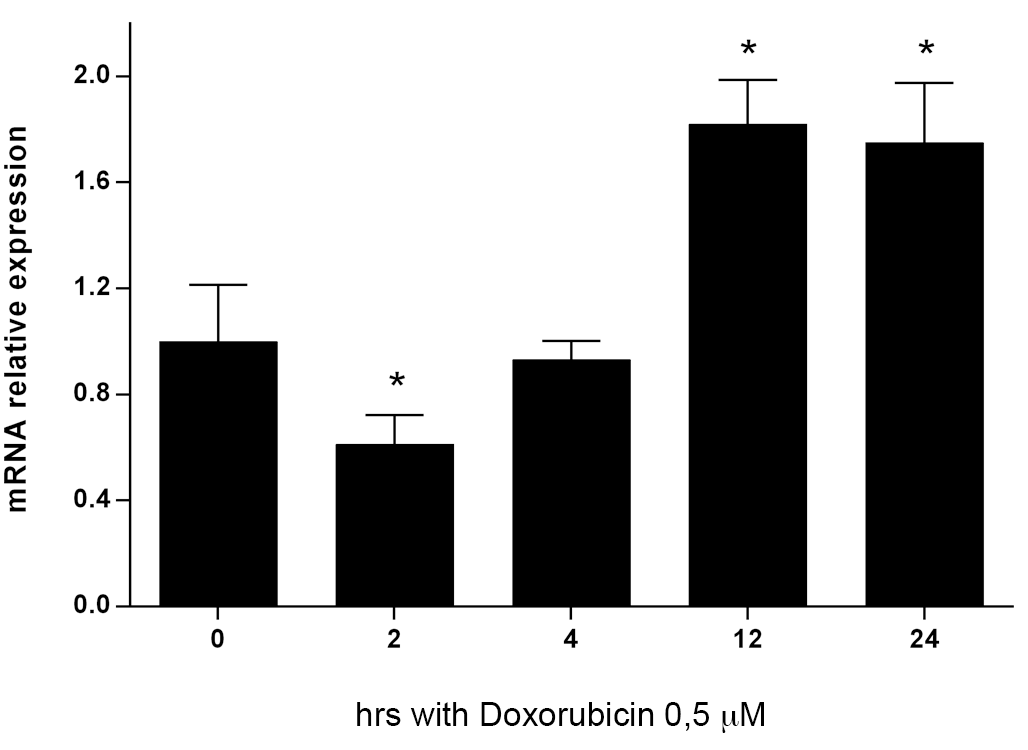


**Supplementary Figure 1**. Dynamic regulation of Sall2mRNA induced by doxorubicin treatment**.** *Sall2 ^+/+^* MEFs were exposed to 0.5 μM doxorubicin for various times. Sall2 mRNA expression was measured by quantitative real-time PCR (qPCR) relative to *cyclophilin A.* Sall2 mRNA expression at time 0 was defined as 1. Values from triplicate samples are representative of two independent experiments. Error bars, mean +/- SD; *p< 0,05 for decrease or increase of Sall2 relative to t=0 hrs.
