# Supplementary material for: Sall2 is required for proapoptotic Noxa expression and genotoxic stress-induced apoptosis by doxorubicin
Source: Cell Death Dis. 2015 Jul 16;6(7):e1816–. doi: 10.1038/cddis.2015.165 (PMC4650718; doi:10.1038/cddis.2015.165)
Supplement: Supplementary Figure 2 [file cddis2015165x3.doc]

**
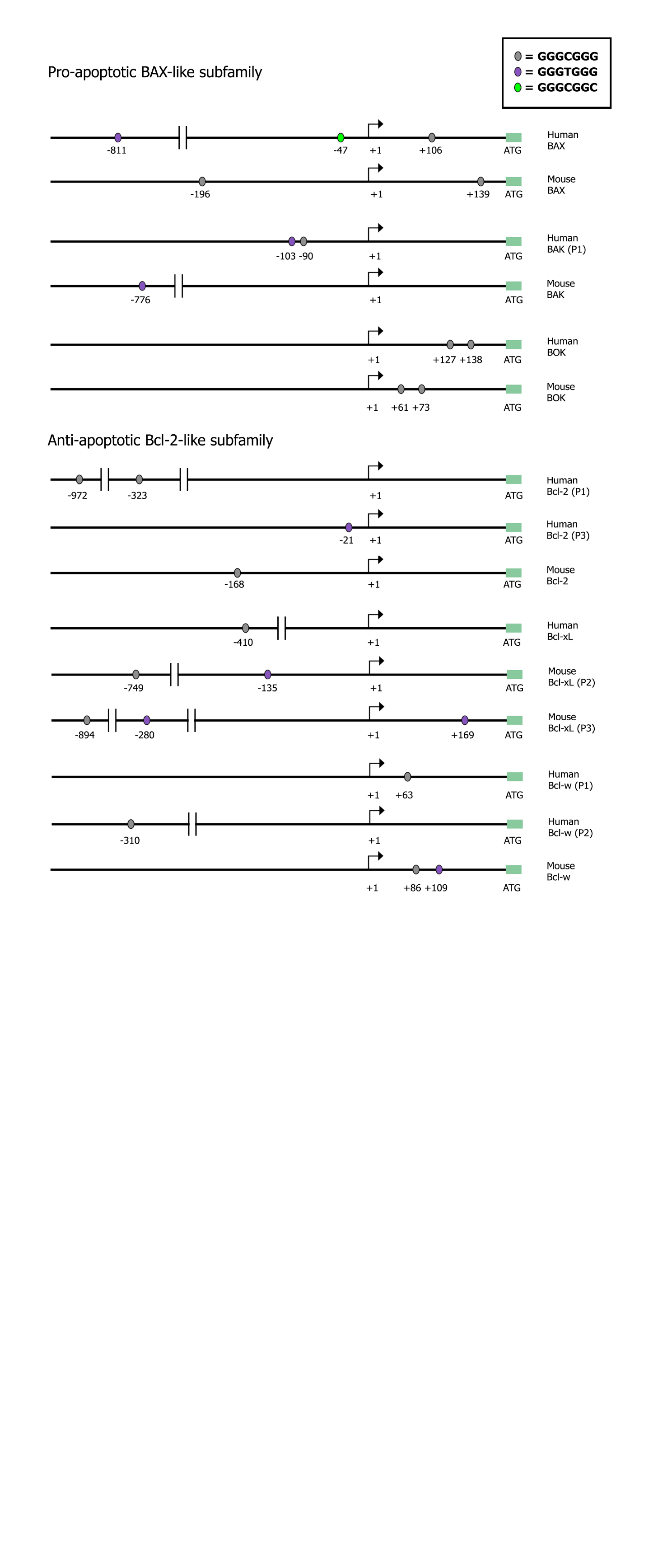

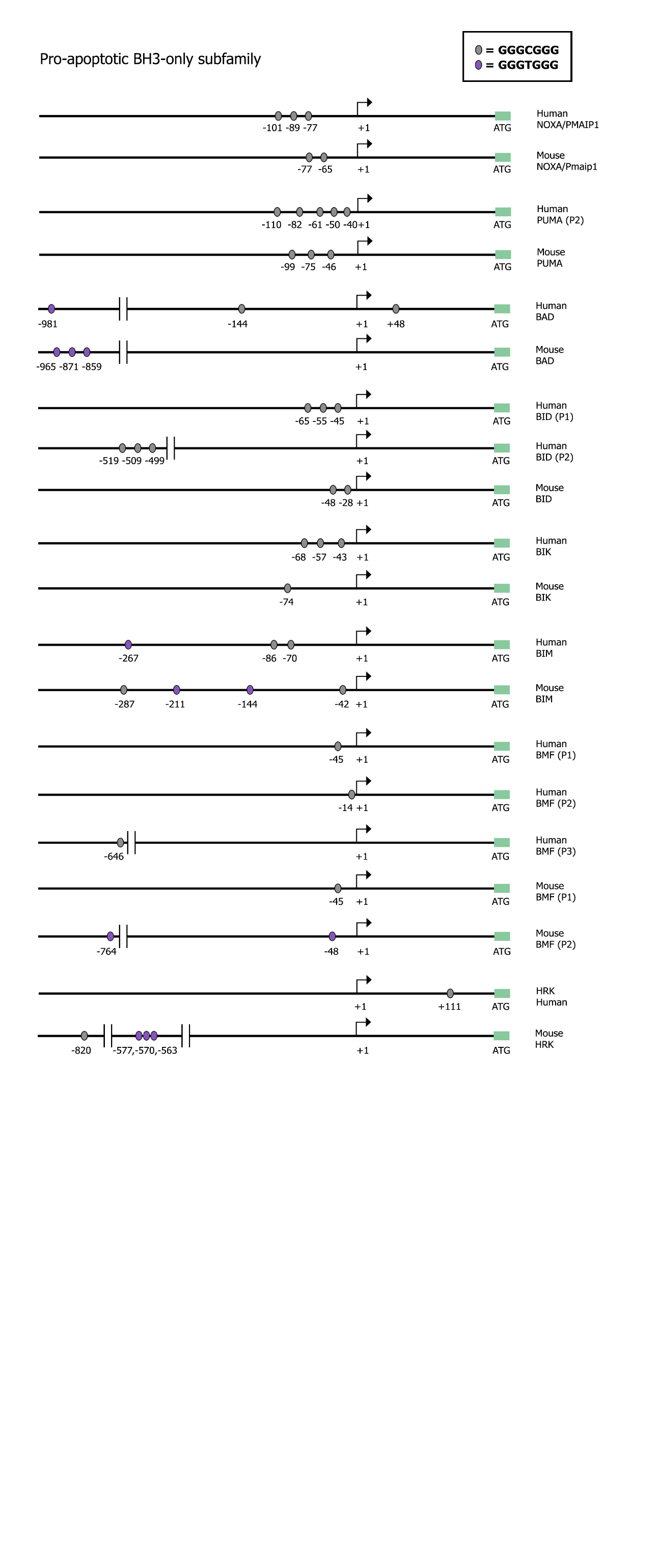
**

**Supplementary Figure 2.** Schematic representation of human and mouse promoter regions from apoptotic-related genes, and the location of putative Sall2 binding sites. Promoter sequences were obtained from The Eukaryotic Promoter Database (<http://epd.vital-it.ch/>) and analyzed for the presence of a previously identified consensus motif (GGGC/TGGG, Gu H, 2011). The Sall2 putative binding sites are represented by gray (GGG**C**GGG), purple (GGG**T**GGG) and green (GGG**C**GG**C**) ovals. The sequences analyzed are from the nucleotides -1000 to the +200 relative to the start site (+1, arrow).
