# Supplementary material for: Sall2 is required for proapoptotic Noxa expression and genotoxic stress-induced apoptosis by doxorubicin
Source: Cell Death Dis. 2015 Jul 16;6(7):e1816–. doi: 10.1038/cddis.2015.165 (PMC4650718; doi:10.1038/cddis.2015.165)
Supplement: Supplementary Figure 3 [file cddis2015165x4.doc]

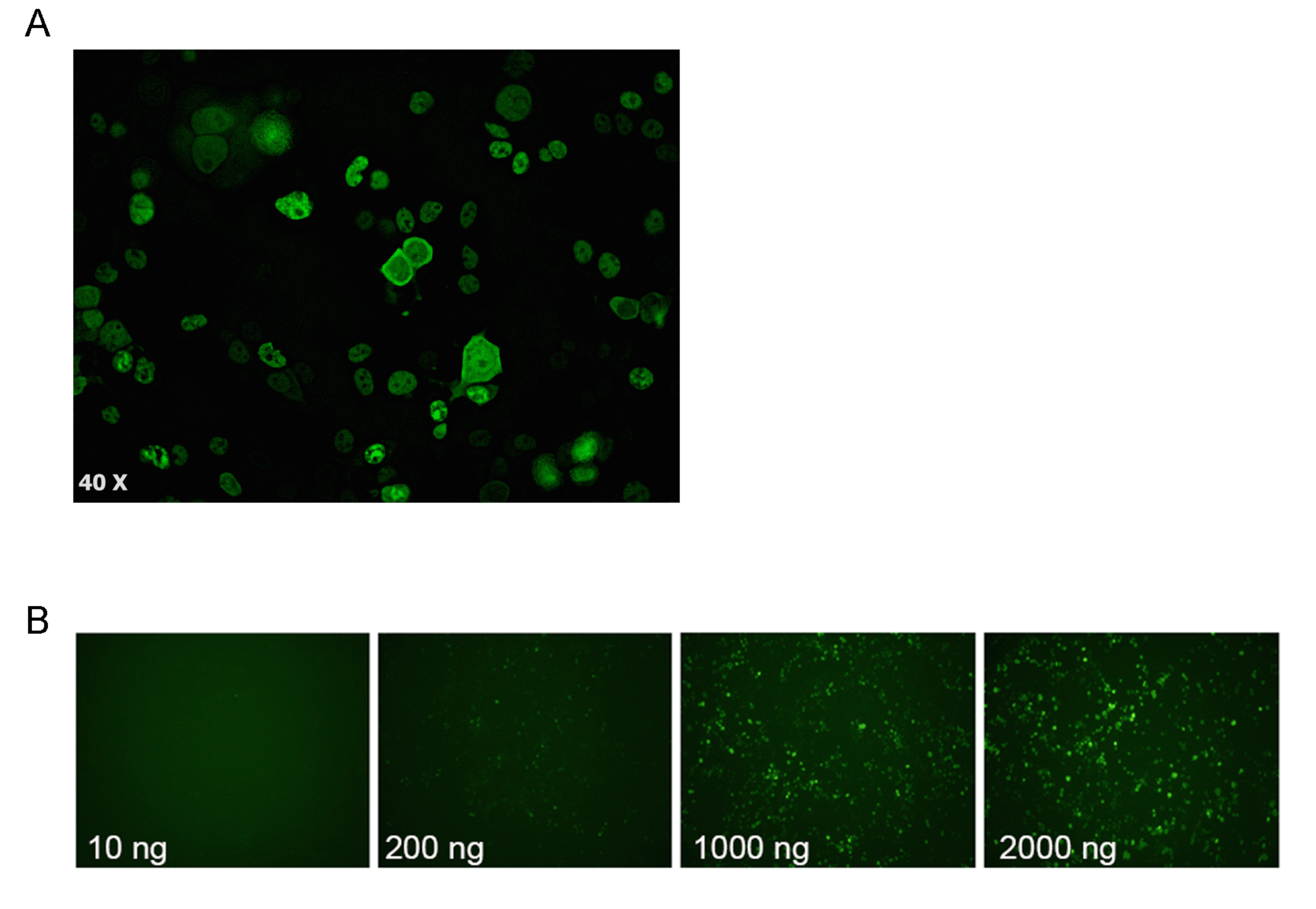


**Supplementary Figure 3.** Detection of Sall2-GFP in HEK 293 cells. After 48 hours of transfection with Sall2-GFP plasmid, cells were observed under fluorescent microscopy. **A**. 40X magnification shows Sall2-GFP mainly in the cell nucleus. **B.** The panel shows increasing green intensity corresponding to increasing Sall2-GFP concentrations (10X magnification).
