# Supplementary material for: Sall2 is required for proapoptotic Noxa expression and genotoxic stress-induced apoptosis by doxorubicin
Source: Cell Death Dis. 2015 Jul 16;6(7):e1816–. doi: 10.1038/cddis.2015.165 (PMC4650718; doi:10.1038/cddis.2015.165)
Supplement: Supplementary Figure 4 [file cddis2015165x5.doc]

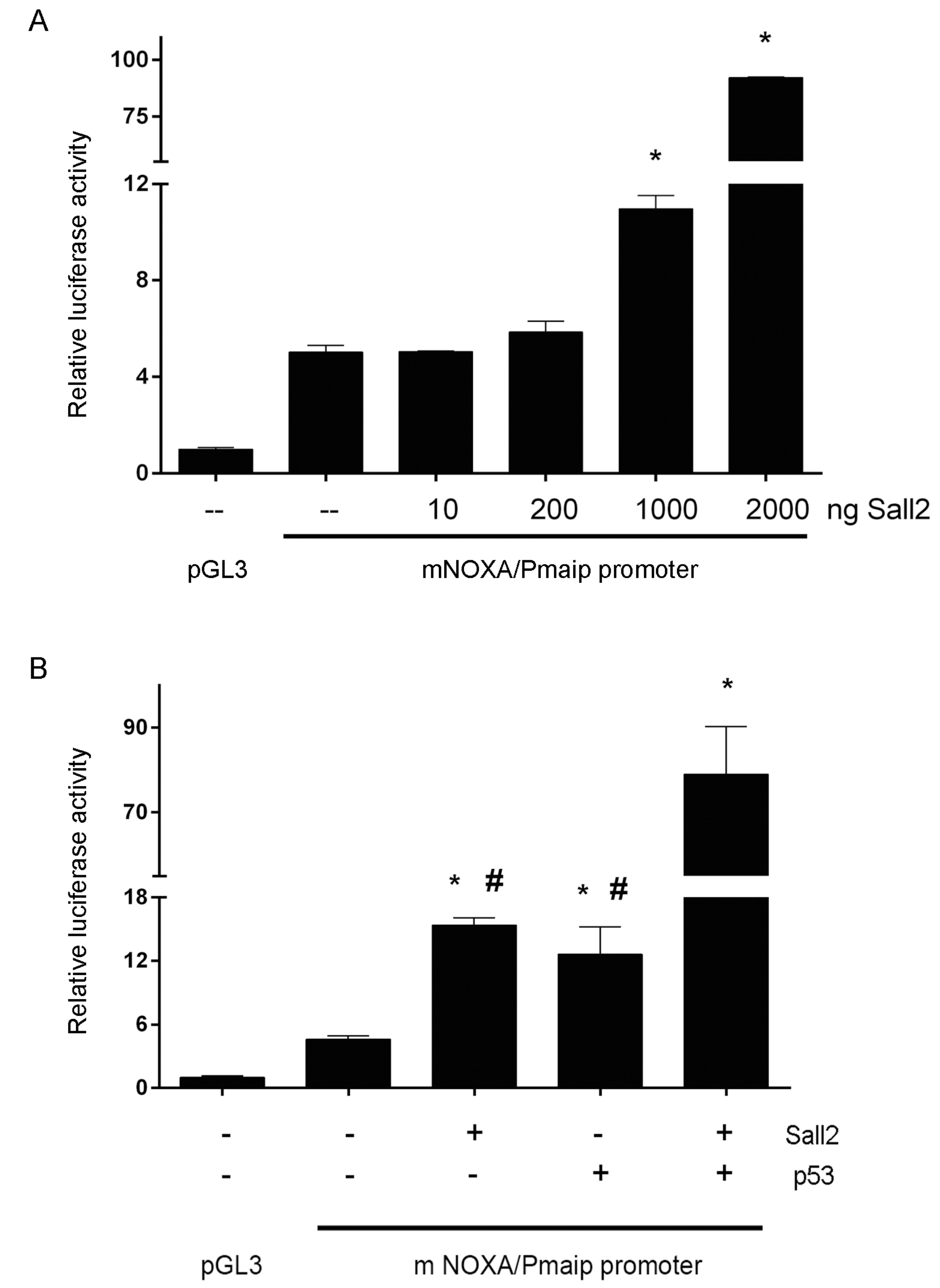


**Supplementary Figure 4.** Sall2 activates *Noxa* promoter independent of p53**. A.** Transient co-transfection of *mNOXA* promoter with increasing concentrations of Sall2 in HCT116 (*p53 null*) cells. **B.** Co-transfection of m*NOXA* promoter with Sall2, p53 or both. Cell extracts were analyzed for luciferase activity and normalized to β-galactosidase activity. Promoter activity is expressed as relative luciferase units to pGL3 empty vector. Results represent three independent experiments performed in triplicate. Each bar represents the mean +/- standard error. Statistic significance was determined by student t-test (* p < 0.05). For figure B, # p< 0.05 relative to Sall2 and p53 together.
