# Supplementary material for: Sall2 is required for proapoptotic Noxa expression and genotoxic stress-induced apoptosis by doxorubicin
Source: Cell Death Dis. 2015 Jul 16;6(7):e1816–. doi: 10.1038/cddis.2015.165 (PMC4650718; doi:10.1038/cddis.2015.165)
Supplement: Supplementary Figure 5 [file cddis2015165x6.doc]

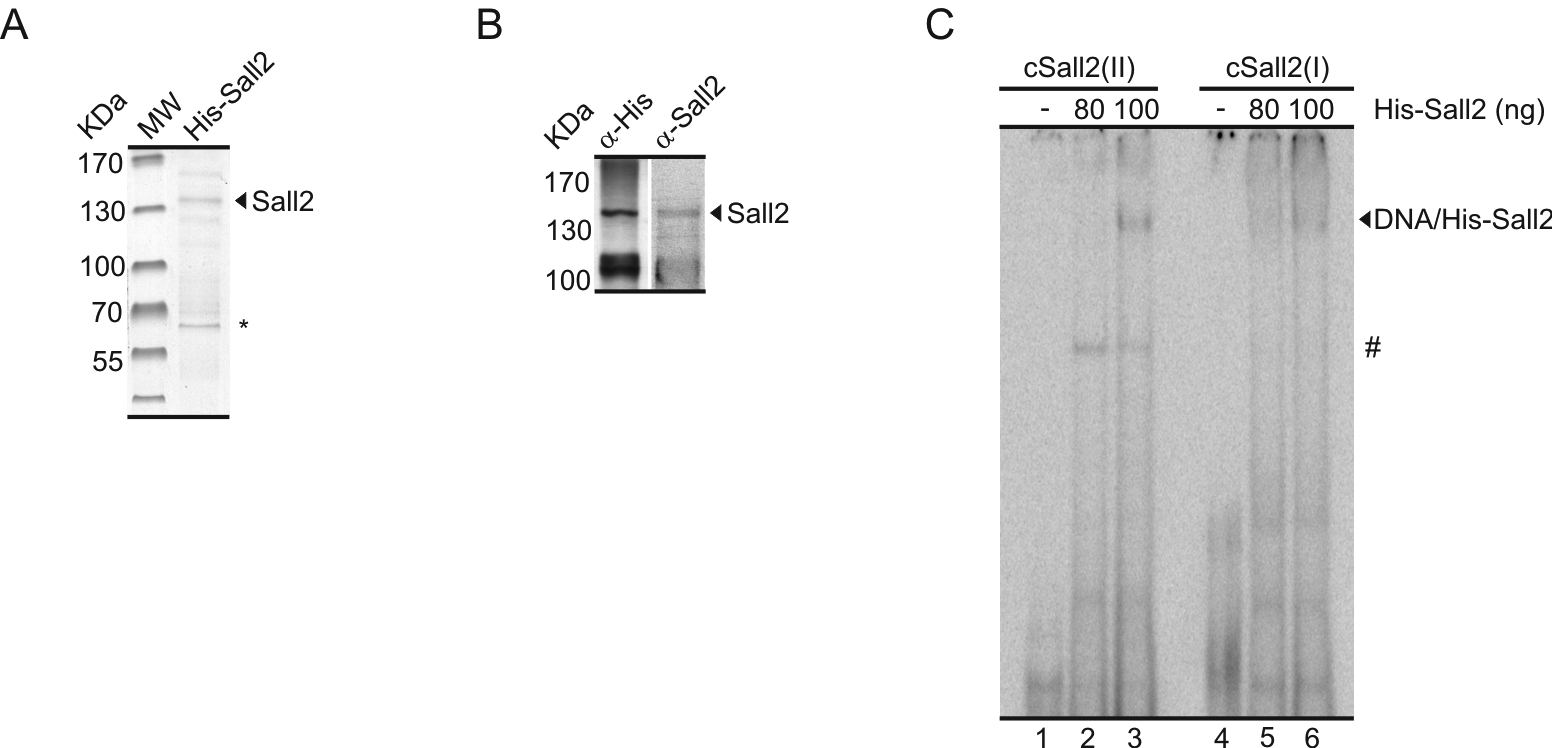


**Supplementary Figure 5.** Purification and binding properties of recombinant His-Sall2 protein. Recombinant His-Sall2 protein was expressed in *E. coli* and then affinity-purified using Ni-NTA agarose resin. **A.** SDS-PAGE analysis of purified His-Sall2 protein. **B.** Western blot analysis of purified His-Sall2 protein. The antibodies used for detection are depicted at the top of the figure. In A and B, the migration of His-Sall2 is indicated at the right side of each figure. **C.** EMSA analysis using recombinant His-Sall2 and two probes, one containing two consensus Sall2 binding sites [cSall2(II)] and the other containing only one [cSall2(I)]. The probes used in each reaction and the amount of His-Sall2 (in ng), are indicated at the top of the figure. Migration of the DNA/His-Sall2 complex is indicated at the right side, where the symbol # represents a minor faster migrating band that appears occasionally when using the cSall2 (II) probe.
