# Supplementary material for: Sall2 is required for proapoptotic Noxa expression and genotoxic stress-induced apoptosis by doxorubicin
Source: Cell Death Dis. 2015 Jul 16;6(7):e1816–. doi: 10.1038/cddis.2015.165 (PMC4650718; doi:10.1038/cddis.2015.165)
Supplement: Supplementary Figure 6 [file cddis2015165x7.doc]

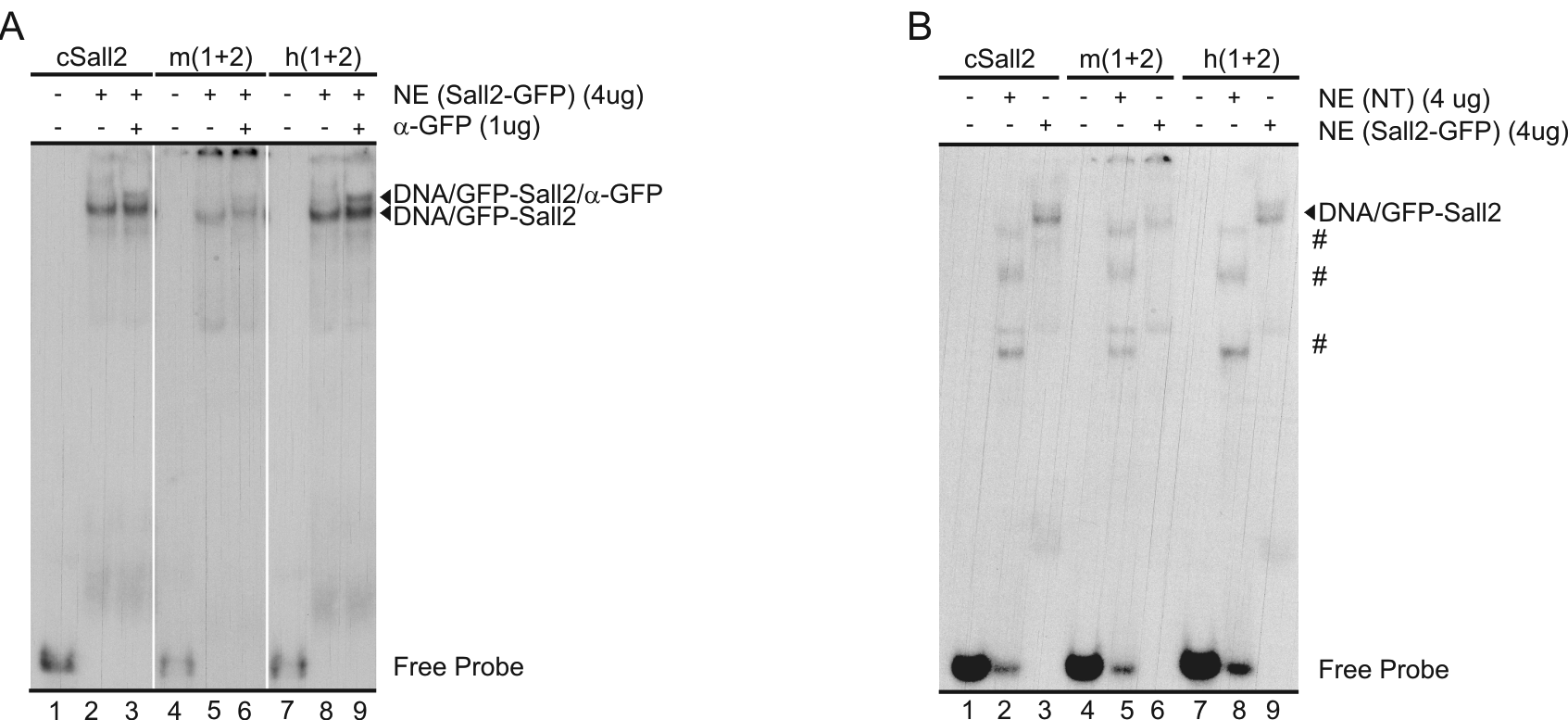


**Supplementary Figure 6**. Identification of the protein-DNA complex generated in EMSAs performed with nuclear extracts containing Sall2-GFP. The assays were performed using nuclear extracts from HEK-293 cells non-transfected [NE (NT)] or transfected with a vector coding for Sall2-GFP [NE (Sall2-GFP)], and probes containing consensus Sall2 binding sites or sites 1+2 of mouse or human *Noxa* gene, as depicted at the top of the figure. **A.** Supershift analysis performed using an anti-GFP antibody. The migration of free probe, DNA/Sall2-GFP and DNA/Sall2-GFP/α-GFP complexes is indicated at the right side of the figure. **B.** Binding patterns of Sall2-GFP-containing and Sall2-GFP-non-containing nuclear extracts. The migration of free probe and DNA/Sall2-GFP complex is indicated at the right side of the figure, the symbol # represents bands observed mainly when using nuclear extracts obtained from non-transfected HEK-293 cells.
