# Supplementary material for: Sall2 is required for proapoptotic Noxa expression and genotoxic stress-induced apoptosis by doxorubicin
Source: Cell Death Dis. 2015 Jul 16;6(7):e1816–. doi: 10.1038/cddis.2015.165 (PMC4650718; doi:10.1038/cddis.2015.165)
Supplement: Supplementary Figure 7 [file cddis2015165x8.doc]

**
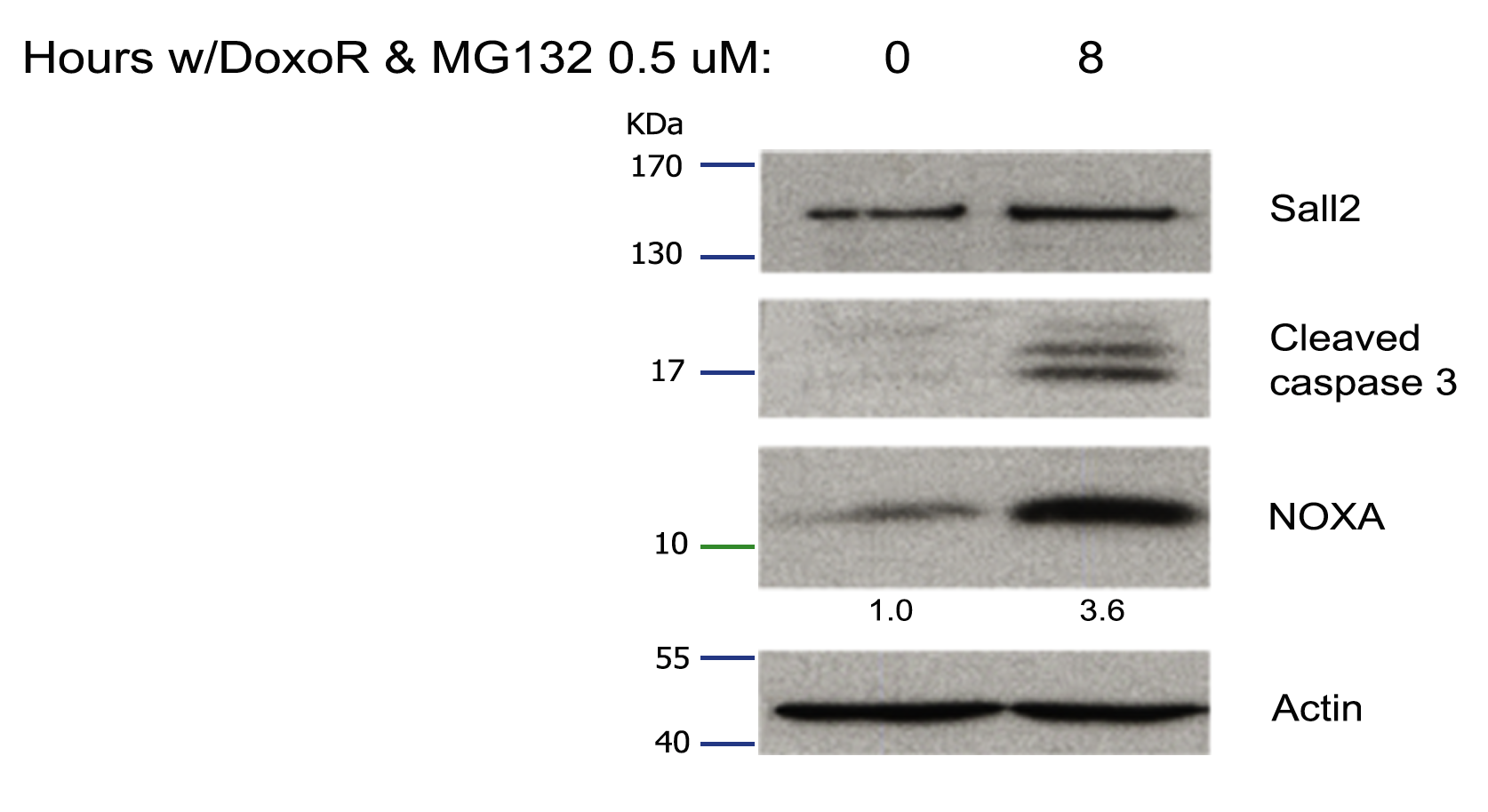
**

**Supplementary Figure 7**. Sall2 and Noxa protein levels are increased in response to doxorubicin + MG132 treatment. Jurkat T cells were treated with DMSO (vehicle) or doxorubicin and MG132 both at 0.5 μM concentration for 8 hours. Cell lysates were collected at each time (0 and 8 hours) and levels of Sall2, cleaved caspase 3 and Noxa were analyzed by Western blot. The ratio of Noxa to actin was measured by densitometry and the fold increase of Noxa over that at time 0 was calculated (values are shown below Noxa Western blot). Actin shows equal loading.
